# Abundance and Diversity of Ophiostomatoid Fungi Associated With the Great Spruce Bark Beetle (*Dendroctonus micans*) in the Northeastern Qinghai-Tibet Plateau

**DOI:** 10.3389/fmicb.2021.721395

**Published:** 2021-10-18

**Authors:** Zheng Wang, Qinzheng Zhou, Guiheng Zheng, Jiaxing Fang, Fuzhong Han, Xingyao Zhang, Quan Lu

**Affiliations:** ^1^Key Laboratory of Forest Protection of National Forestry and Grassland Administration, Research Institute of Forest Ecology, Environment and Protection, Chinese Academy of Forestry, Beijing, China; ^2^Maxiu Forest Farm, Huangnan, China

**Keywords:** bark beetles, conifer, *Endoconidiophora laricicola*, *Leptographium*, *Ophiostoma*, tree pathogens, spruce, symbiosis

## Abstract

The role of several virulent tree pathogens in host death has been overlooked because of the aggressiveness of their associated bark beetles. The great spruce bark beetle (*Dendroctonus micans*) is a widely distributed beetle that infests coniferous plants in Eurasia; however, its associated fungi have been poorly studied. Therefore, in this study, we elucidated the diversity of ophiostomatoid fungi associated with *D. micans* in the northeastern Qinghai-Tibet Plateau through field investigation, laboratory isolation, and culture analyses. A total of 220 strains of ophiostomatoid fungi were isolated from adults and tunnel galleries of *D. micans* infesting *Picea crassifolia.* We identified that the isolated strains belonged to eight ophiostomatoid species, including five new species (*Ophiostoma huangnanense* sp. nov., *Ophiostoma maixiuense* sp. nov., *Ophiostoma sanum* sp. nov., *Leptographium sanjiangyuanense* sp. nov., and *Leptographium zekuense* sp. nov.), one undefined species (*Ophiostoma* sp. 1), and two known species (*Ophiostoma bicolor* and *Endoconidiophora laricicola*), using phylogenetic analysis of multigene DNA sequences and morphological characteristics. This is the first time that *E. laricicola*, a pioneer invader and virulent pathogen, has been reported in China. We found that *E. laricicola* was the dominant species, accounting for 40.91% of the total number of ophiostomatoid communities. This study enriched the knowledge of the fungal associates of *D. micans* and elucidated that it carried the virulent pathogen *E. laricicola* at a surprisingly high frequency. Our findings show increased species association between *D. micans* and ophiostomatoid fungi and provide a basis for understanding the occurrence of forest diseases and pests.

## Introduction

Global climate change has led to an increase in forest diseases and pest outbreaks, as well as an increased risk of the formation of new associations between forest pests and pathogens, resulting from globalization (Wingfield et al., 2015, 2016, 2017; Biedermann et al., 2019). Consequently, increasing attention is being paid to insect-borne forest pathogens and their vectors, including several tree pathogens belonging to the ophiostomatoid fungi and their bark beetle vectors due to their intimate association and devastating effects on forests (Eckhardt and Menard, 2013; Hofstetter et al., 2015; Wingfield et al., 2016, 2017; Biedermann and Vega, 2020).

Ophiostomatoid fungi belong to the class Sordariomycetes of the phylum Ascomycota; they are the members of the orders Ophiostomatales (Sordariomycetidae) and Microascales (Hypocreomycetidae) (De Beer and Wingfield, 2013). They include some of the most devastating tree pathogens that are associated with bark beetles, such as *Ophiostoma novo-ulmi* subspecies *novo-ulmi* and *O. novo-ulmi* subspecies *americana* associated with the scolytid bark beetles, which are responsible for the Dutch elm disease—one of the most devastating tree diseases in the world (Brasier, 1991; Kirisits, 2013; Miyamoto et al., 2019; Hessenauer et al., 2020); *Leptographium wageneri* vectored by the root-feeding bark beetles, which causes black stain root disease of conifers (Harrington and Cobb, 1988; Eckhardt, 2013), and *Raffaelea lauricola* transmitted by the redbay ambrosia beetle, which is a lethal pathogen for the trees of the Lauraceae family in North America (Fraedrich et al., 2008).

Because of the damaging effects of the beetles, the mortality of the conifers is often attributed to beetle infestations, and the role of fungi is overlooked. Ophiostomatoid fungi associated with several well-known conifer specialists have been shown to transmit virulent-tree pathogens, such as *Grosmannia clavigera* associated with *Dendroctonus ponderosae* (Solheim and Krokene, 1998; Rice et al., 2007; Alamouti et al., 2011), *Endoconidiophora rufipenni* associated with *Dendroctonus rufipennis* (Solheim and Safranyik, 1997; Wingfield et al., 1997), *Leptographium qinglingense* associated with *Dendroctonus armandi* (Tang and Chen, 1999; Tang et al., 2004), and *Leptographium procerum* associated with *Dendroctonus valens* (Lu Q. et al., 2009; Marincowitz et al., 2020). The genus *Dendroctonus* contains 20 species, including many conifer killers (Armendáriz-Toledano et al., 2015; Six and Bracewell, 2015; Godefroid et al., 2019). However, after more than a century of research, the association between fungi and *Dendroctonus* spp. remains understudied (Six and Bracewell, 2015).

In China, there are currently two known native species of *Dendroctonus*, *D. armandi* and *Dendroctonus micans*, and one invasive species from Central and North America, *D. valens* (Yin et al., 1984; Huang and Lu, 2015). *Dendroctonus valens* killed millions of native pine trees in China since its introduction in the 1990s (Li et al., 2001; Yan et al., 2005; Sun et al., 2013). Therefore, their associated ophiostomatoid fungi have been studied in detail in both their native and invasive regions (Lu et al., 2008; Lu M. et al., 2009; Lu Q. et al., 2009; Taerum et al., 2013; Marincowitz et al., 2020). To date, a total of 32 ophiostomatoid fungi have been reported to be associated with *D. valens*, among which the virulent tree pathogen *L. procerum* is considered to be a major contributing factor to host tree death and plays a role in the destructive impact of this beetle in China (Lu et al., 2010, 2011). *L. procerum* was originally considered to have been introduced into China from North America along with *D. valens* (Lu et al., 2011; Sun et al., 2013; Taerum et al., 2013). However, population genetic analysis suggests that it probably originated from Europe and arrived independently in China, where the fungus and the beetle formed an association (Taerum et al., 2017). This suggests that when a fungus is accidentally introduced into a new environment, it can associate with new vectors and become an important pathogen (Wingfield et al., 2016, 2017; Marincowitz et al., 2020). Studies on ophiostomatoid fungi associated with *D. armandi* are few, with no more than six known species recorded, most of which are undefined (Tang et al., 2004; Hu et al., 2015; Skelton et al., 2018).

*Dendroctonus micans* primarily attacks spruce trees and is thought to be a native of Siberia; it spread to West Asia and Europe over several decades, mainly through the transport of infested logs and host planting (Grégoire, 1988; Meurisse et al., 2008; Six and Bracewell, 2015; Godefroid et al., 2019). However, the beetle was once thought to have no symbiotic fungi (Six and Bracewell, 2015). Before 2016, only one study showed the association of fungi with this beetle; the study reported the association of *Ophiostoma canum* with *D. micans* in Europe (Lieutier et al., 1992). Subsequently, a total of 26 strains representing six ophiostomatoid fungi (*Ophiostoma ainoae*, *Ophiostoma micans*, *Ophiostoma nitidum*, *Ophiostoma qinghaiense*, *Ophiostoma shangrilae*, and *Ophiostoma tetropii*) were reported, successively, to be associated with the beetle, two of which (*O. nitidum* and *O. tetropii*) were associated only with the mites present on this beetle (Yin et al., 2016; Chang et al., 2020).

The objective of this study was to elucidate the diversity of ophiostomatoid fungi associated with *D. micans* through field investigation, laboratory isolation, and culture analyses. We conducted accurate species identification through phylogenetic analysis of multigene DNA datasets combined with morphological characteristics. Our findings will increase the understanding of the potential role of ophiostomatoid fungal pathogens in the infection of *D. micans* within a tree host.

## Materials and Methods

### Sample Collection and Isolation

**FIGURE 1:**
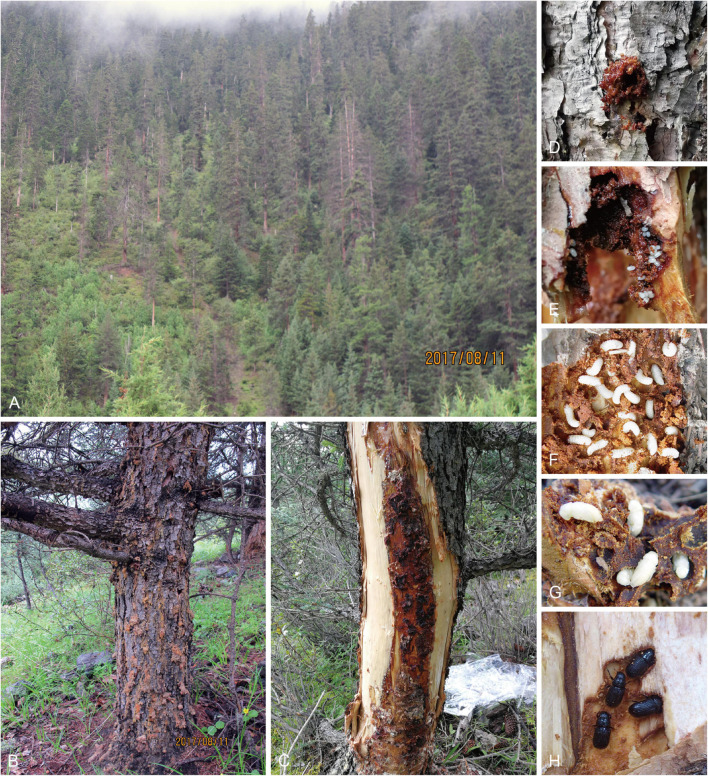
Damage to spruce by *D. micans* and ophiostomatoid fungi at landscape level **(A)** and tree level **(B,C)**. Pioneer beetle invading spruce with obvious signs of reddish globules of resin tubes showing the vigorousness and active defense of the host **(D)**. The life cycle of the *D. micans* in galleries on spruce: egg **(E)**, larva **(F)**, pupa **(G)**, and adult **(H)**.

Adult beetles and their galleries were collected during the mass flight period from three sites (Figure 1 and Supplementary Table 1) in the northeastern Qinghai-Tibet Plateau in Qinghai Province, China, from 2019 to 2020. Twenty adults and their galleries were collected at each sampling site, placed in sterile Eppendorf tubes and envelope bags, and stored at 4°C until fungal isolation. Each adult was dismembered into approximately 30 pieces and transferred onto 2% water agar. The surface of the galleries was disinfected using 1.5% sodium hypochlorite for 1 min, rinsed with sterile water three times, cut into approximately 3 × 3 mm^2^ tissue pieces, and transferred onto 2% water agar. After incubation in the dark for a period of time, mycelium apex and/or single-spore isolation was conducted to purify all strains. Pure culture was transferred onto 2% malt extract agar (MEA) for growth. The representative strains of each morphotype were selected for subsequent studies based on the initial analysis of their macroscopic and microscopic characteristics. All strains were deposited in the culture collection at the Forest Pathology Laboratory at the Chinese Academy of Forestry (CXY). The representative strains were deposited at the China Forestry Culture Collection Center (CFCC).

### Morphological Studies

An Olympus SZX16 stereomicroscope (Olympus, Center Valley, PA, United States), Zeiss Axio Imager A2 microscope, and a Zeiss Axiocam 506 color digital camera (Carl Zeiss Ltd., Munich, Germany) were used to observe and record the morphological structures of the isolated fungi. For each holotype, the lengths and widths of 30 reproductive structures were measured and presented in the following format: (minimum–) (mean–standard deviation) – (mean + standard deviation) (–maximum). All relevant data from the type specimens were deposited in MycoBank^1^.

The following culturing conditions were used for the morphological studies: a 5 mm diameter agar plug from an actively growing margin of fungal colonies was placed in the center of a 90-mm-diameter Petri plate containing 2% MEA, and five replicate plates for each species were incubated in the dark at temperatures ranging from 0 to 40°C at 5°C intervals. Two colony diameters, perpendicular to each other, were measured every 24 h, until the mycelium reached the margin of the Petri dish.

### DNA Extraction, Polymerase Chain Reaction Amplification, and Sequencing

A sufficient amount of actively growing mycelia from the colony margin of each representative strain was collected for DNA extraction, which was conducted using an Invisorb Spin Plant Mini Kit (Tiangen, Beijing, China), following the manufacturer’s instructions. Primer pairs ITS1-F/ITS4 (White et al., 1990; Gardes and Bruns, 1993), ITS3/LR3 (Vilgalys and Hester, 1990; White et al., 1990), Bt1a/Bt1b (Glass and Donaldson, 1995), Bt2a/Bt2b (Glass and Donaldson, 1995), EF1F/EF2R (Jacobs et al., 2004), CL2F/CL2R (Duong et al., 2012), and Algr52_412-433_f1/Algr52_1102_1084_r1 (Stielow et al., 2015) were used for the amplification of the internal transcribed spacer regions 1 and 2 of the nuclear ribosomal DNA operon, including the 5.8S region (ITS), the internal transcribed spacer 2 and part of the 28S of the rDNA operon (ITS2-LSU), the β-tubulin gene region (Tub1 and Tub2), the transcription elongation factor 1-α gene region (TEF1-α), the calmodulin gene region (CAL), and the partial 60S ribosomal protein RPL10 gene (60S), respectively.

Polymerase chain reaction (PCR) assays were conducted using the 2 × Taq PCR MasterMix (Tiangen, Beijing, China), following the manufacturer’s instructions. The PCR conditions used for the amplification of the seven regions are as follows: an initial denaturation step at 95°C for 3 min, followed by 35 cycles of 1 min at 95°C, 45 s at 54–58°C, and 1 min at 72°C, and a final chain elongation at 72°C for 8 min. The PCR products were sequenced by two-directional sequencing using the primers specified above by Majorbio Co., Ltd., (Shanghai, China).

### Phylogenetic Analysis

The preliminary identities of the representative strains in this study were elucidated by comparing their morphological characteristics and conducting a standard nucleotide BLAST search using their nucleotide sequences. Reference sequences in the phylogenetic analyses were downloaded from GenBank. Alignments of the sequences were performed with MAFFT online v.7 (Katoh et al., 2019) using the FFT-NS-i strategy with a 200 PAM/k = 2 scoring matrix, a gap opening penalty of 1.53, and an offset value of 0.00. Datasets were further edited manually using Molecular Evolutionary Genetic Analyses 7.0 (Kumar et al., 2016). The edited datasets were then used for phylogenetic analysis using maximum likelihood (ML), maximum parsimony (MP), and Bayesian inference (BI) methods.

Maximum likelihood analyses were performed using RAxML-HPC v.8.2.3 (Stamatakis, 2014) with the GTR + G model of site substitution, including estimation of gamma-distributed rate heterogeneity and a proportion of invariant sites (Stamatakis, 2006). Finally, a maximum number of 1,000 trees were retained, and 1,000 bootstrap replicates were performed to estimate bootstrap support values.

Maximum parsimony analyses were performed using PAUP^∗^ version 4.0b10 (Swofford, 2003) with a heuristic search option of 1,000 random addition sequences. The gaps were treated as the fifth base. Clades compatible with the 50% majority rule in the bootstrap consensus tree were retained. The following settings were used: tree bisection reconnection branch swapping, starting tree obtained *via* stepwise addition, steepest descent not in effect, and MulTrees effective.

Bayesian inference analyses were performed using MrBayes v. 3.1.2 (Ronquist and Huelsenbeck, 2003) with the best substitution models for each dataset determined using the corrected Akaike information criterion (AICc) in jModelTest v.2.1.7 (Darriba et al., 2012). Four Markov chain Monte Carlo chains were run simultaneously from a random starting tree for 5,000,000 generations with a sampling frequency of every 100 generations to calculate posterior probabilities. The first 25% of the sampled trees were discarded as burn-in, and the remaining trees were used to calculate the posterior probabilities.

Phylogenetic trees were edited using FigTree v.1.4.3^2^ and Adobe Illustrator CS6. The final alignments were deposited in TreeBASE (No. S28319).

## Results

### Sample Collection and Isolation

A total of 220 strains of ophiostomatoid fungi were isolated from 60 adults and 240 tissue pieces from *D. micans* galleries within the *Picea crassifolia* host. Among them, 147 strains were isolated from adult beetles and 73 from their galleries (Supplementary Table 2). Fifty-three strains were selected as representative strains based on their macroscopic and microscopic morphological features (Supplementary Table 1). Standard nucleotide BLAST searches in GenBank showed that these strains belonged to the genera *Ophiostoma*, *Leptographium*, and *Endoconidiophora* (Supplementary Table 1).

### Phylogenetic Analysis

For every individual sequence dataset, similar topologies with slight variations in the node support values were generated using the three phylogenetic methods. The best-fit evolutionary models were obtained using jModelTest v. 2.1.7 (Supplementary Table 3). Phylogenetic analyses showed that the 53 representative strains belonged to eight taxa. Five taxa belonged to the genus *Ophiostoma* (Taxa 1–5), two taxa belonged to the genus *Leptographium* (taxons 6 and 7), and one taxon belonged to the genus *Endoconidiophora* (taxon 8).

#### Ophiostoma

**FIGURE 2:**
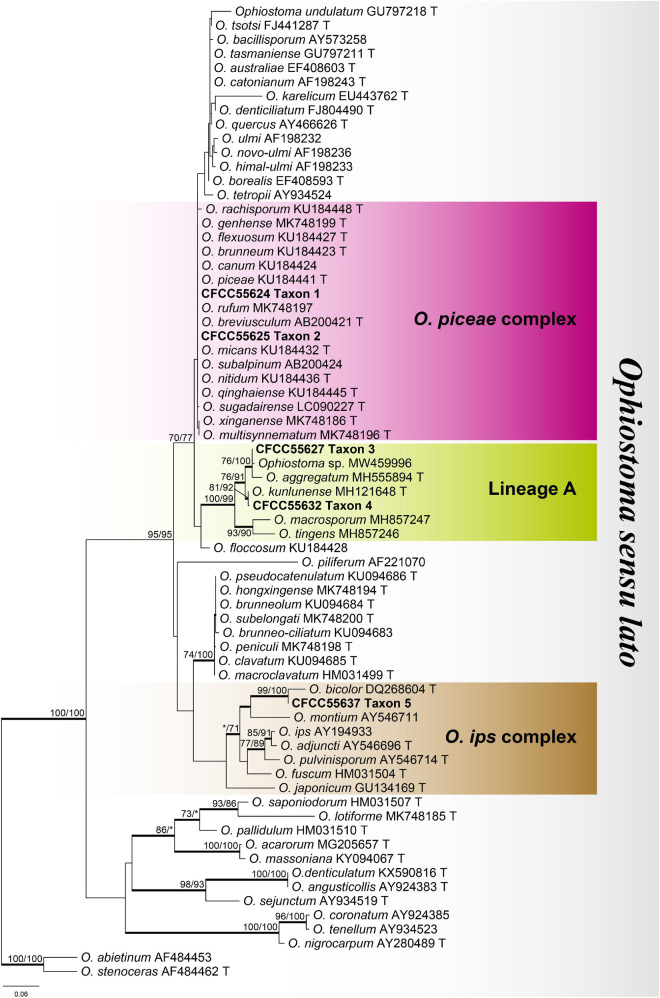
Phylogram of *Ophiostoma sensu lato* based on ITS sequence data. Bold branches indicate posterior probability values ≥ 0.9. The MP/ML bootstrap support values ≥ 70% are recorded at the nodes. T, ex-type isolates.

**FIGURE 3:**
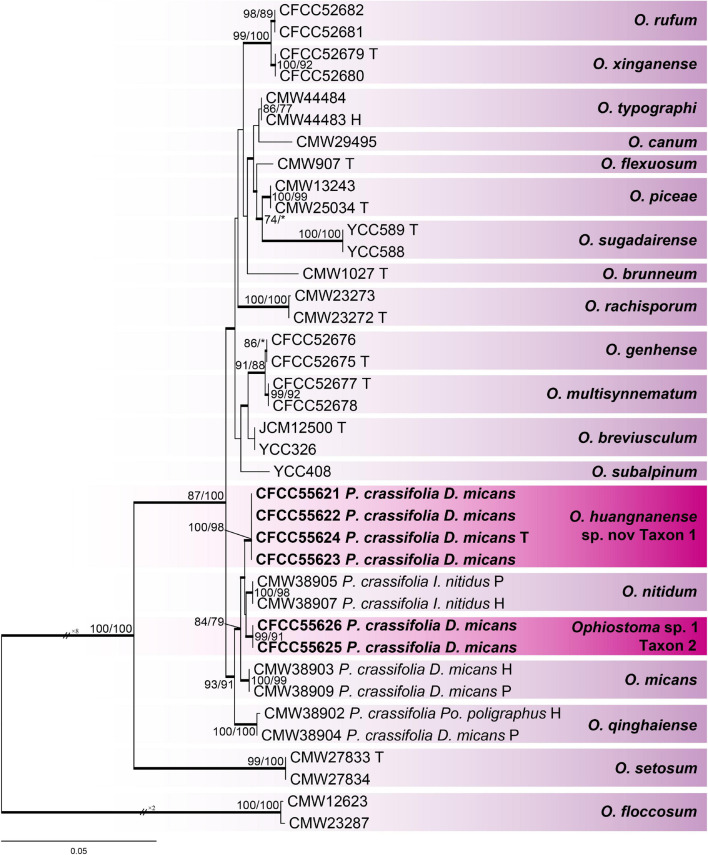
Phylogram of *Ophiostoma piceae* complex (including taxons 1 and 2) based on combined (Tub2 + TEF1-α + CAL) sequence data. Bold branches indicate posterior probability values ≥ 0.9. The MP/ML bootstrap support values ≥ 70% are recorded at the nodes. *D*., *Dendroctonus*; *P*., *Picea*; *Po*., *Polygrphus*; H, ex-holotype; P, ex-paratype; T, ex-type isolates.

**FIGURE 4:**
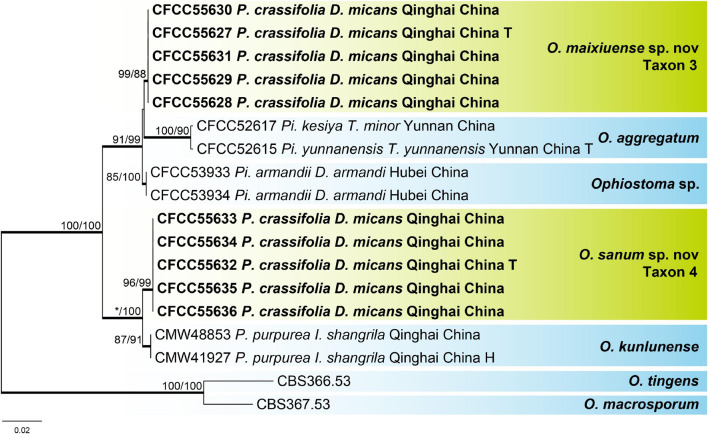
Phylogram of lineage A (including taxons 3 and 4) based on combined (ITS + Tub2) sequence data. Bold branches indicate posterior probability values ≥ 0.9. The MP/ML bootstrap support values ≥ 70% are recorded at the nodes. *D*., *Dendroctonus*; *I*., *Ips*; *P*., *Picea*; *Pi*., *Pinus*; *T*., *Tomicus*; T, ex-type isolates.

The ITS dataset of *Ophiostoma* included 69 sequences representing 68 taxa and 684 characters, including gaps. Our strains belonged to the *Ophiostoma piceae* complex, lineage A, and the *Ophiostoma ips* complex (Figure 2). Among them, six representative strains nested in the *O. piceae* complex (Figure 3 and Supplementary Figures 1–3), 10 representative strains nested in lineage A (Figure 4 and Supplementary Figures 4, 5), and 4 in the *O. ips* complex (Supplementary Figures 6–8).

For the phylogenetic analysis of *O. piceae* complex, Tub2, TEF1-α, CAL, and combined (Tub2 + TEF1-α + CAL) datasets were used, which contained 401, 1,016, 866, and 2,283 characters, respectively, including gaps. Our six representative strains within this complex formed two clades with high node support values, taxons 1 and 2, which were most closely related to *Ophiostoma nitidum*, *O. micans*, and *O. qinghaiense*, based on the results of the combined dataset phylogenetic analysis (Figure 3). The phylograms of the Tub2 and CAL datasets (Supplementary Figures 1, 3) showed that taxon 1 was an independent clade with high branch support values. In contrast, an unstable phylogenetic placement was elucidated in taxon 2: taxon 2 and *O. micans* in the phylograms of Tub2 (Supplementary Figure 1), taxons 1 and 2 in the phylograms of TEF1-α (Supplementary Figure 2), and taxon 2 and *O. qinghaiense* (Supplementary Figure 3) were not separated from each other, respectively.

For the phylogenetic analysis of lineage A, the ITS, Tub2, and combined (ITS + Tub2) datasets were used, which contained 677, 447, and 1,124 characters, respectively, including gaps. Our 10 representative strains within this lineage formed two clades with high node support values, presented as taxons 3 and 4 and were most closely related to *Ophiostoma aggregatum* and *Ophiostoma kunlunense*, respectively, based on the results of the combined dataset phylogenetic analysis (Figure 4). The phylograms of the ITS and Tub2 datasets (Supplementary Figures 4, 5) showed that taxons 3 and 4 were independent clades with high node support values, respectively.

For the phylogenetic analysis of *O. ips* complex, the ITS, Tub2, and combined (ITS + Tub2) datasets were used, which contained 673, 430, and 1,103 characters, respectively, including gaps. Our four representative strains within this complex formed a clade together with *Ophiostoma bicolor*, which is presented as taxon 5 (Supplementary Figures 6–8).

#### Leptographium

**FIGURE 5:**
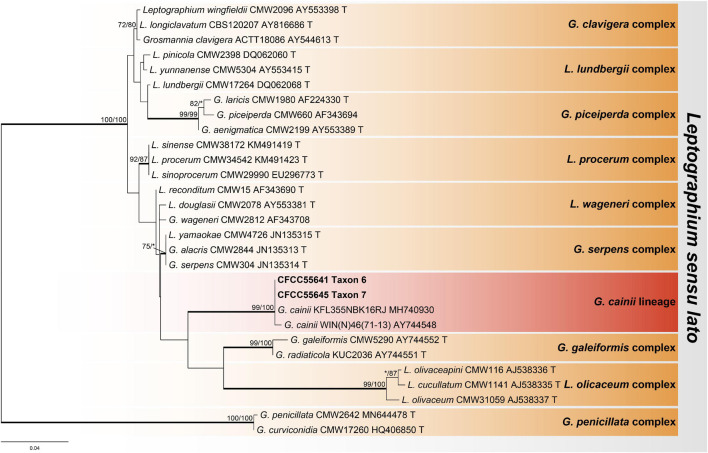
Phylogram of *Leptographium sensu lato* based on ITS2-LSU sequence data. Bold branches indicate posterior probability values ≥ 0.9. The MP/ML bootstrap support values ≥ 70% are recorded at the nodes. T, ex-type isolates.

**FIGURE 6:**
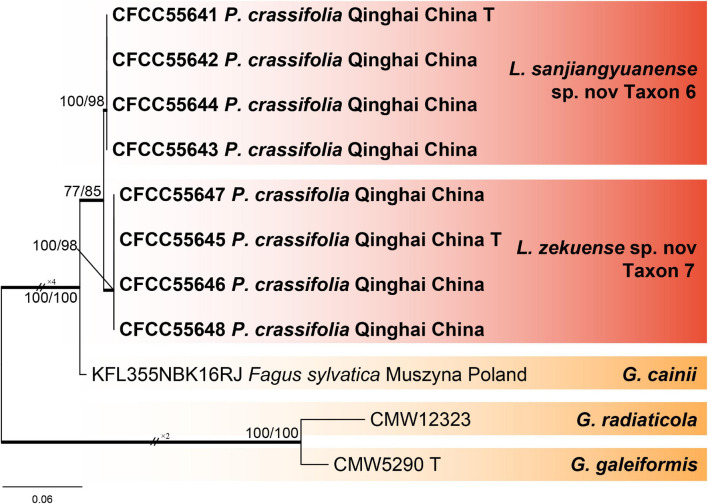
Phylogram of *G. cainii* lineage (including taxons 6 and 7) based on combined (Tub2 + TEF1-α) sequence data. Bold branches indicate posterior probability values ≥ 0.9. The MP/ML bootstrap support values ≥ 70% are recorded at the nodes. *P*., *Picea*; T, ex-type isolates.

The ITS2-LSU dataset of *Leptographium* included 29 sequences representing 28 taxa and 582 characters, including gaps. Our strains belonged to the *Grosmannia cainii* lineage (Figure 5), and we used Tub2, TEF1-α, and combined (Tub2 + TEF1-α) datasets for phylogenetic analysis, which contained 403, 915, and 1,318 characters, respectively, including gaps. Our eight representative strains within this lineage formed two clades with high node support values, which are presented as taxons 6 and 7, and were most closely related to *G. cainii* (Figure 6 and Supplementary Figures 9, 10).

#### Endoconidiophora

**FIGURE 7:**
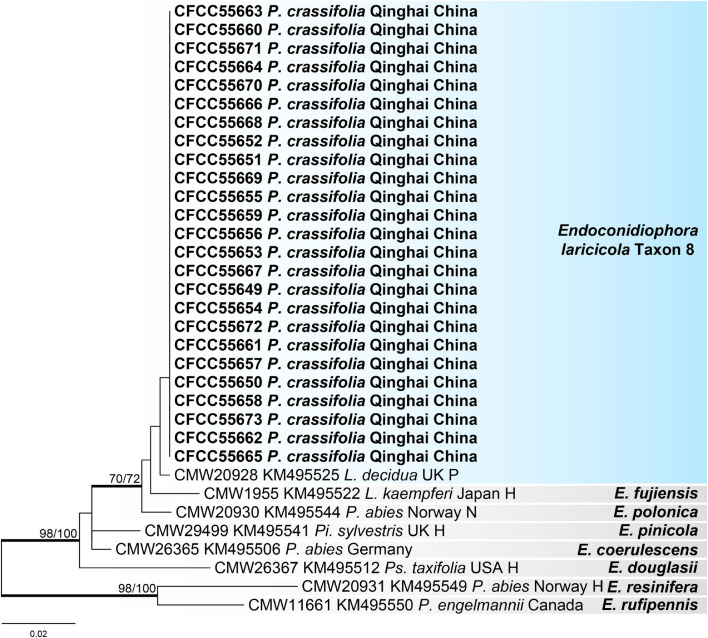
Phylogram of *Endoconidiophora* (including taxon 8) based on 60S sequence data. Bold branches indicate posterior probability values ≥ 0.9. The MP/ML bootstrap support values ≥ 70% are recorded at the nodes. *L*., *Larix*; *P*., *Picea*; *Pi*., *Pinus*; *Ps*., *Pseudotsuga*; H, ex-holotype; N, ex-neotype; P, ex-paratype.

For the phylogenetic analysis of genus *Endoconidiophora*, the 60S and Tub1 datasets were used, which contained 393 and 537 characters, respectively, including gaps. Our 25 representative strains within this genus formed a clade together with *Endoconidiophora laricicola*, which is presented as taxon 8 (Figure 7 and Supplementary Figure 11).

### Taxonomy

**FIGURE 8:**
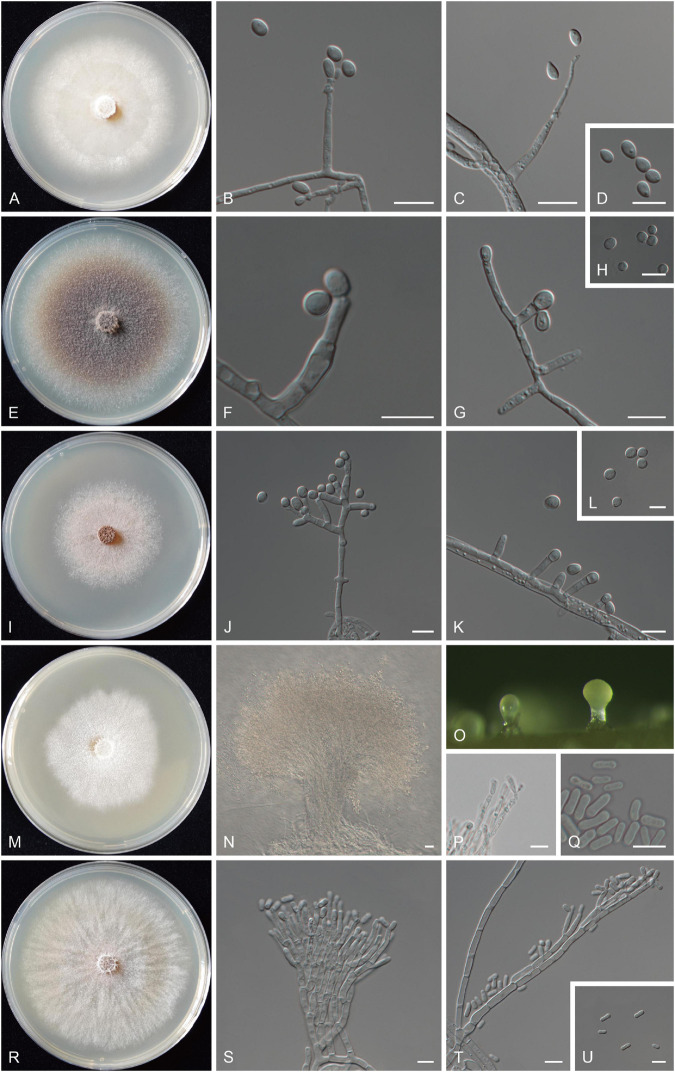
Morphological characteristics of the five new species. **(A–D)** Morphological characteristics of *O. huangnanense* sp. nov. (taxon 1, CXY3004, holotype). **(A)** Fourteen-day-old cultures on 2% MEA. **(B–D)** Sporothrix-like asexual morph: conidiogenous cells and conidia. **(E–H)** Morphological characteristics of *O. maixiuense* sp. nov. (taxon 3, CXY3007, holotype) **(E)** Seven-day-old cultures on 2% MEA. **(F–H)** Hyalorhinocladiella-like asexual morph: conidiogenous cells and conidia. **(I–L)** Morphological characteristics of *Ophiostoma sanus* sp. nov. (taxon 4, CXY3012, holotype). **(I)** Seven-day-old cultures on 2% MEA. **(J–L)** Hyalorhinocladiella- to raffaelea-like asexual morph: conidiogenous cells and conidia. **(M–Q)** Morphological characteristics of *L. sanjiangyuanense* sp. nov. (taxon 6, CXY3021, holotype). **(M)** Ten-day-old cultures on 2% MEA. **(N,O)** Pesotum-like asexual morph. **(P,Q)** Conidiogenous cells of pesotum-like asexual morph and conidia. **(R–U)** Morphological characteristics of *L. zekuense* sp. nov. (taxon 7, CXY3025, holotype). **(R)** Ten-day-old cultures on 2% MEA. **(S–U)** Hyalorhinocladiella-like asexual morph: conidiogenous cells and conidia. Scale bars: panels **(B–D,F–H,J–L,P,Q,S–U)** = 10 μm; panel **(N)** = 20 μm.

***Ophiostoma huangnanense*** Z. Wang and Q. Lu, sp. nov. (Figures 8A–D)

MycoBank MB 839882.

Type: China, Qinghai Province, Huangnan City, Zeku County, from *D. micans* infesting *P. crassifolia*, Aug. 2019, Z. Wang and Q. Z. Zhou, holotype CXY3004, ex-type culture CFCC55624.

Etymology: The epithet huangnanense (Latin) refers to the city of Huangnan, from where this fungus was collected.

Diagnosis: Phylogenetically sister to *O. nitidum*, differs by asexual morph (sporothrix-like vs. pesotum-like).

Description: Sexual morph not observed. Asexual morph: sporothrix-like.

Sporothrix-like morph: conidiogenous cells arising directly from hyphae (6.4–) 11.2–27.1 (−35.2) × (1.7–) 2.0–2.9 (−3.7) μm. Conidia hyaline, smooth, ovate or obovoid, aseptate (4.7–) 5.0–6.8 (−8.0) × (2.6–) 2.9–3.7 (−3.9) μm.

Cultures: Colonies on 2% MEA at 25°C reaching a diameter of 66 mm in 15 days, initially hyaline, later becoming pure white, then becoming brownish or olivaceous from the center of the colony to the sides, mycelium superficial with many aerial mycelia, and the colony margin thinning radially. Optimal temperature for growth is 20°C; no growth was observed at 0°C and 35°C.

Associated insects: *D. micans.*

Known host and distribution: Known on *P. crassifolia* in Qinghai, China.

Notes: Phylogenetic analysis revealed that *O. huangnanense* and *Ophiostoma* sp. 1 formed a lineage with three species (*O. nitidum*, *O. micans*, and *O. qinghaiense*), which were also isolated from *P. crassifolia* in Qinghai (Figure 3; Yin et al., 2016). *O. huangnanense* can be distinguished from the other three species by the presence of a sporothrix-like asexual state, which is absent in the latter. In terms of culture characteristics on 2% MEA, the optimal growth temperatures of *O. huangnanense*, *O. nitidum*, *O. micans*, and *O. qinghaiense* were 20, 25, 25, and 20°C, respectively (Yin et al., 2016). At 20°C, *O. huangnanense* shows radial growth faster than that reported for *O. qinghaiense* (6.4 vs. 3 mm/d, Yin et al., 2016).

Additional specimens examined: China, Qinghai Province, Huangnan City, Zeku County, from *D. micans* infesting *P. crassifolia*, Aug. 2019, Z. Wang and Q. Z. Zhou, culture CFCC55621, CFCC55622, and CFCC55623.

***Ophiostoma maixiuense*** Z. Wang and Q. Lu, sp. nov. (Figures 8E–H)

MycoBank MB 839883.

Type: China, Qinghai Province, Huangnan City, Zeku County, from *D. micans* infesting *P. crassifolia*, Aug. 2019, Z. Wang and Q. Z. Zhou, holotype CXY3007, ex-type culture CFCC55627.

Etymology: The epithet maixiuense (Latin) refers to the Maixiu forest farms from where this fungus was collected.

Diagnosis: Phylogenetically sister to *O. aggregatum*, differs by asexual morph (hyalorhinocladiella-like vs. leptographium-like).

Description: Sexual morph not observed. Asexual morph: hyalorhinocladiella-like.

Hyalorhinocladiella-like asexual morph: conidiogenous cells arising directly from aerial hyphae (5.3–) 6.6–10.9 (−13.4) × (2.2–) 2.5–3.2 (−3.4) μm. Conidia hyaline, smooth, oval or ovate, aseptate (4.7–) 5.1–6.7 (−7.4) × (4.4–) 4.5–5.6 (−6.0) μm.

Cultures: Colonies on 2% MEA at 25°C reaching a diameter of 78 mm in 7 days, cottony, initially hyaline, later becoming brown from the center of the colony to the sides, mycelium superficial with many aerial mycelia, and the colony margin thinning radially. Optimal temperature for growth is 25°C; no growth was observed at 0°C and 35°C.

Associated insects: *D. micans.*

Known host and distribution: Known on *P. crassifolia* in Qinghai, China.

Notes: *O. maixiuense* is closely related to *O. aggregatum* (Wang et al., 2019). The collection sites, hosts, and insect vectors of the two species were different. The former was isolated from *D. micans* infesting *P. crassifolia* in Qinghai, China. The latter was isolated from *Tomicus* beetles infesting *Pinus* in Yunnan, China (Wang et al., 2019).

Additional specimens examined: China, Qinghai Province, Huangnan City, Zeku County, from *D. micans* infesting *P. crassifolia*, Aug. 2019, Z. Wang and Q. Z. Zhou, culture CFCC55628, CFCC55629, CFCC55630, and CFCC55631.

***Ophiostoma sanum*** Z. Wang and Q. Lu, sp. nov. (Figures 8I–L)

MycoBank MB 839884.

Type: China, Qinghai Province, Huangnan City, Zeku County, from *D. micans* infesting *P. crassifolia*, Aug. 2019, Z. Wang and Q. Z. Zhou, holotype CXY3012, ex-type culture CFCC55632.

Etymology: The name is based on the hope that everyone stays healthy (Latin: sanum) in the context of the current COVID-19 pandemic.

Diagnosis: Phylogenetically sister to *O. kunlunense*, differs by asexual morph (hyalorhinocladiella- to raffaelea-like vs. pesotum-like).

Description: Sexual morph not observed. Asexual morph: hyalorhinocladiella- to raffaelea-like.

Hyalorhinocladiella- to raffaelea-like morph: conidiogenous cells arising directly from aerial hyphae (5.2–) 7.0–10.7 (−12.0) × (2.3–) 2.6–3.2 (−3.4) μm. Conidia hyaline, smooth, oval or ovate, and aseptate (4.5–) 4.8–6.3 (−7.3) × (4.3–) 4.4–5.2 (−5.9) μm.

Cultures: Colonies on 2% MEA at 25°C reaching a diameter of 50 mm in 7 days, hyaline to white gray initially, later becoming gray brown; mycelium superficial with sparsely aerial mycelia, and the colony margin irregular. Optimal temperature for growth is 25°C; no growth was observed at 0°C and 35°C.

Associated insects: *D. micans.*

Known host and distribution: Known on *P. crassifolia* in Qinghai, China.

Notes: *O. sanum* and *O. kunlunense* were the closest in phylogenetic analysis, and both were collected from Qinghai, China (Chang et al., 2020). However, their substrates were different. The former was isolated from *D. micans* infesting *P. crassifolia*, while the latter was isolated from *Ips shangrila* infesting *Picea purpurea*. *O. sanum* can be distinguished from *O. kunlunense* based on differences in the optimal growth temperature (25°C vs. 20°C). Furthermore, *O. sanum* grows slowly at 5°C and 30°C, but *O. kunlunense* cannot grow under these temperatures. *O. maixiuense* and *O. sanum* belonged to lineage A and were the most closely related to *O. aggregatum*, *Ophiostoma* sp., and *O. kunlunense* (Figure 4 and Supplementary Figures 4, 5). All five species were isolated from conifers in China, but the host and vector species were different, viz. *O. maixiuense* and *O. sanum* associated with *D. micans* infesting *P. crassifolia*, *O. aggregatum* associated with *Tomicus* infesting *Pinus*, *Ophiostoma* sp. associated with *D. armandi* infesting *Pinus armandii*, and *O. kunlunense* associated with three mites in the galleries of *I. shangrila* infesting *P. purpurea* (Figure 4; Wang et al., 2019; Chang et al., 2020).

Additional specimens examined: China, Qinghai Province, Huangnan City, Zeku County, from *D. micans* infesting *P. crassifolia*, Aug. 2019, Z. Wang and Q. Z. Zhou, culture CFCC55633, CFCC55634, CFCC55635, and CFCC55636.

***Leptographium sanjiangyuanense*** Z. Wang and Q. Lu, sp. nov. (Figures 8M–Q)

MycoBank MB 839885.

Type: China, Qinghai Province, Huangnan City, Zeku County, from *D. micans* infesting *P. crassifolia*, Aug. 2019, Z. Wang and Q. Z. Zhou, holotype CXY3021, ex-type culture CFCC55641.

Etymology: The epithet sanjiangyuanense (Latin) refers to the Sanjiangyuan National Nature Reserve, from where this fungus was collected.

Diagnosis: Phylogenetically sister to *Leptographium zekuense*, differs by asexual morph (pesotum-like vs. hyalorhinocladiella-like).

Description: Sexual morph not observed. Asexual morph: pesotum-like.

Pesotum-like morph: synnemata solitary (182–) 213–298 (−337) μm tall, including the conidiogenous apparatus, the base hyaline (30.9–) 39.5–67.3 (−78.7) μm wide. Conidiogenous cells (15.7–) 17.3–23.4 (−26.1) × (1.6–) 1.8–2.3 (−2.7) μm. Conidia hyaline, smooth, clavate to cylindrical, aseptate (6.4–) 7.0–8.9 (−10.3) × (2.6–) 2.7–3.2 (−3.6) μm.

Cultures: Colonies on 2% MEA at 25°C reaching a diameter of 53 mm in 10 days, hyaline initially, later becoming pure white, mycelium superficial with many aerial mycelia, and the colony margin irregular. Optimal temperature for growth is 20°C; no growth was observed at 0°C and 35°C.

Associated insects: *D. micans.*

Known host and distribution: Known on *P. crassifolia* in Qinghai, China.

Notes: *L. sanjiangyuanense* and *L. zekuense* are the closest in phylogenetic analysis, and both were isolated from *D. micans* infesting *P. crassifolia* in Qinghai, China. The former can be distinguished from the latter by the differences in the optimal growth temperature (20°C vs. 25°C). Furthermore, at the 25°C growth temperature, *L. sanjiangyuanense* showed a radial growth slower than *L. zekuense* (5.3 vs. 7.3 mm/d, Figures 8M,R). Culture characteristics: colonies of *L. sanjiangyuanense* on 2% MEA at 25°C are pure white with irregular margins, but *L. zekuense* colonies are yellowish to olivaceous with margin thinning radially (Figures 8M,R).

Additional specimens examined: China, Qinghai Province, Huangnan City, Zeku County, from *D. micans* infesting *P. crassifolia*, Aug. 2019, Z. Wang and Q. Z. Zhou, culture CFCC55642, CFCC55643, and CFCC55644.

***Leptographium zekuense*** Z. Wang and Q. Lu, sp. nov. (Figures 8R–U)

MycoBank MB 839886.

Type: China, Qinghai Province, Huangnan City, Zeku County, from *D. micans* infesting *P. crassifolia*, Aug. 2019, Z. Wang and Q. Z. Zhou, holotype CXY3025, ex-type culture CFCC55645.

Etymology: The epithet zekuense (Latin) refers to Zeku County, from where this fungus was collected.

Diagnosis: See comparisons between *L. sanjiangyuanense* and *L. zekuense* under *L. sanjiangyuanense*.

Description: Sexual morph not observed. Asexual morph: hyalorhinocladiella-like.

Hyalorhinocladiella-like morph: conidiogenous cells arising directly from aerial hyphae (9.9–) 15.4–23.8 (−30.1) × (1.7–) 1.8–2.3 (−2.4) μm. Conidia hyaline, smooth, oval to elliptical, aseptate (5.0–) 6.3–7.7 (−8.2) × (2.3–) 2.5–3.1 (−3.4) μm.

Cultures: Colonies on 2% MEA at 25°C reaching a diameter of 73 mm in 10 days, cottony, initially yellowish, later becoming olivaceous from the colony margin to center, mycelium superficial with many aerial mycelia, and the colony margin thinning radially. Optimal temperature for growth is 25°C; no growth was observed at 0 and 35°C.

Associated insects: *D. micans.*

Known host and distribution: Known on *P. crassifolia* in Qinghai, China.

Notes: *L. zekuense*, *L. sanjiangyuanense*, and *G. cainii* combined formed the *G. cainii* lineage, which was distinct from other known species complex in *Leptographium sensu lato* (De Beer and Wingfield, 2013). *G. cainii* is not often found, and the type strain of this species has been isolated from *Picea mariana* in Manitoba, Canada (Olchowecki and Reid, 1974). Recently, a new strain of *G. cainii* was isolated from *Fagus sylvatica* in Muszyna, Poland (Jankowiak et al., 2019). This lineage was enriched by the discovery of two new species in this study.

Additional specimens examined: China, Qinghai Province, Huangnan City, Zeku County, from *D. micans* infesting *P. crassifolia*, Aug. 2019, Z. Wang and Q. Z. Zhou, culture CFCC55646, CFCC55647, and CFCC55648.

## Discussion

In this study, a total of 220 ophiostomatoid fungal strains representing eight species were obtained from adults and galleries of *D. micans* infesting *P. crassifolia* in the northeastern Qinghai-Tibet Plateau. The results of this study significantly increased the number of eight ophiostomatoid fungal species reported to be associated with *D. micans* from seven (Lieutier et al., 1992; Yin et al., 2016; Chang et al., 2020) to a total of 15 species. Among the eight species reported in this study, three species are new (*O. huangnanense*, *O. maixiuense*, and *O. sanum*), one is undefined (*Ophiostoma* sp. 1 due to a relatively small number of strains and an unstable phylogenetic placement in this taxon), and one is a known species (*O. bicolor*) belonging to *Ophiostoma*; two are new species (*L. sanjiangyuanense* and *L. zekuense*) belonging to *Leptographium*; and one is a known species (*E. laricicola*) belonging to *Endoconidiophora*. *E. laricicola* was the dominant species in this study, followed by *L. sanjiangyuanense*, *O. huangnanense*, *O. sanum*, *O. bicolor*, *O. maixiuense*, *L. zekuense*, and *Ophiostoma* sp. 1, which accounted for 40.91, 16.36, 10.91, 10.91, 8.18, 6.82, 4.55, and 1.36% of the total ophiostomatoid fungal strains, respectively (Supplementary Table 2).

None of the seven species reported in previous studies were found in this study. Among them, *O. canum* has only been reported once in Europe (Lieutier et al., 1992); only few strains of *O. ainoae*, *O. micans*, *O. qinghaiense*, and *O. shangrilae* were found in China (Yin et al., 2016); *O. nitidum* (eight strains) and *O. tetropii* (three strains) were only found to be associated with the mites present on *D. micans* (Chang et al., 2020). Therefore, these seven previously reported ophiostomatoid fungi may be occasional species associated with *D. micans*. Furthermore, it also indicates that a large number of ophiostomatoid fungi that are associated with *D. micans* are yet to be discovered.

In this investigation, the five species of the genus *Ophiostoma* belong to two species complexes (*O. piceae* complex and *O. ips* complex) and one lineage (lineage A). *O. huangnanense* and *Ophiostoma* sp. 1 are the members of the *O. piceae* complex, which contains many species isolated from a variety of bark beetles infesting coniferous trees in China in recent years (Yin et al., 2016; Chang et al., 2019, 2020; Wang et al., 2019, 2020). The clade of *O. huangnanense* had high node support values in the phylogenetic analysis of the four datasets (Tub2, TEF1-α, CAL, and the combined of Tub2 + TEF1-α + CAL), whereas the clade of *Ophiostoma* sp. 1 had low node support values and unstable positions in the phylogenetic analysis of different datasets (Figure 3 and Supplementary Figures 1–3). In addition, we only obtained three strains of *Ophiostoma* sp. 1; hence, it was recorded as an undefined species.

*Ophiostoma bicolor* belongs to the *O. ips* complex and is widely distributed in North America and Eurasia (Davidson, 1955; Yamaoka et al., 1997; Kirisits, 2004; Alamouti et al., 2007; Jankowiak et al., 2017; Chang et al., 2019, 2020). It is mainly associated with *Ips* beetles that infest different types of spruce and is considered a pathogen for the host (Solheim, 1988; Christiansen and Solheim, 1994). In this study, for the first time, the association between *O. bicolor* and *D. micans* was established.

A newly recorded species in China, *E. laricicola*, was the dominant species associated with *D. micans* infesting *P. crassifolia* in this study, which is different from the insect vectors (*Ips cembrae*) and hosts (*Larix decidua*) previously reported in Europe (Redfern et al., 1987; Stauffer et al., 2001). We found that our strains were genetically differentiated from the European strains as per the 60S phylogenetic tree (Figure 7), but they were intertwined in the Tub1 phylogenetic tree (Supplementary Figure 11). Therefore, more gene fragments or genetic loci should be analyzed to infer their phylogenetic relationships, origin, evolution, and transmission routes.

*Endoconidiophora* was first separated from *Ceratocystis* through a multi-locus phylogenetic analysis of the 60S ribosomal protein RPL10 (60S), the nuclear ribosomal DNA large subunit (LSU), and mini-chromosome maintenance complex component 7 (MCM7) in 2014 (De Beer et al., 2014). This genus currently contains eight species, many of which are well-known plant pathogens and exhibit stable association features with their respective vectors and hosts. The most well-known paradigms are the association of *Ips typographus*–*Endoconidiophora polonica*–*Picea abies*, *I. cembrae*–*E. laricicola*–*L. decidua*, and *Ips subelongatus*–*Endoconidiophora fujiensis*–*Larix kaempferi*. All three *Endoconidiophora* species are pioneer invaders and the most virulent fungal associates (Redfern et al., 1987; Solheim, 1988; Yamaoka et al., 1998; Yamaoka, 2017). Among them, *E. polonica* can utilize host rich phenolic defense substances as carbon source, which is considered to be an important reason for its virulence (Wadke et al., 2016). However, limited studies have been done to elucidate the pathogenic mechanisms of *E. laricicola* and *E. fujiensis*. *E. laricicola* is only known to cause larch dieback and death (Redfern et al., 1987), and *E. fujiensis* can kill 30-year-old Japanese larch after artificial inoculation (Yamaoka et al., 1998; Yamaoka, 2017).

Interestingly, *I. cembrae* was originally thought to be a widely distributed species, and Stauffer et al. (2001) reported that it represented two allopatric species, *I. cembrae* and *I. subelongatus*, which was inferred from its DNA sequences and fungal associates. In parallel, *E. fujiensis* (originally believed to be *E. laricicola*), associated with *I. subelongatus*, was also separated from *E. laricicola* (Marin et al., 2005). In addition, *E. fujiensis* has been shown to exhibit different virulence in different hosts (Liu, 2015; Wang et al., 2020). Therefore, the pathogenicity of virulent *E. laricicola* in native and potential hosts should be studied urgently. Furthermore, the vector and host of *E. laricicola*, as well as the reasons for the geographical change in distribution, need further elucidation.

In general, a total of eight ophiostomatoid fungi associated with *D. micans* were found in this study, which indicates that *D. micans*, widely distributed in Eurasia, may have rich associated mycobiota that is still unexplored. Moreover, the elucidation of the pathogenicity of these fungi is the next urgent step in assessing their role in conifer mortality.

## Data Availability Statement

The original contributions presented in the study are included in the article/Supplementary Material, further inquiries can be directed to the corresponding author/s.

## Author Contributions

QL, XZ, and ZW designed the study. QZ, FH, and JF collected the samples. ZW, QZ, and GZ performed DNA extraction and PCR amplification. ZW performed the research and analyzed the data. QL and ZW wrote the manuscript. All authors reviewed and approved the final manuscript.

## Conflict of Interest

The authors declare that the research was conducted in the absence of any commercial or financial relationships that could be construed as a potential conflict of interest.

## Publisher’s Note

All claims expressed in this article are solely those of the authors and do not necessarily represent those of their affiliated organizations, or those of the publisher, the editors and the reviewers. Any product that may be evaluated in this article, or claim that may be made by its manufacturer, is not guaranteed or endorsed by the publisher.

## Footnotes

1
www.MycoBank.org

2
http://tree.bio.ed.ac.uk/software/figtree/

## Supplementary Material

Supplementary Figure 1 Phylogram of *Ophiostoma piceae* complex (including taxons 1 and 2) based on Tub2 sequence data. Bold branches indicate posterior probability values ≥ 0.9. The MP/ML bootstrap support values ≥ 70% are recorded at the nodes. H, ex-holotype; P, ex-paratype; T, ex-type isolates.

Supplementary Figure 2 Phylogram of *Ophiostoma piceae* complex (including taxons 1 and 2) based on TEF1-α sequence data. Bold branches indicate posterior probability values ≥ 0.9. The MP/ML bootstrap support values ≥ 70% are recorded at the nodes. H, ex-holotype; P, ex-paratype; T, ex-type isolates.

Supplementary Figure 3 Phylogram of *Ophiostoma piceae* complex (including taxons 1 and 2) based on CAL sequence data. Bold branches indicate posterior probability values ≥ 0.9. The MP/ML bootstrap support values ≥ 70% are recorded at the nodes. H, ex-holotype; P, ex-paratype; T, ex-type isolates.

Supplementary Figure 4 Phylogram of Lineage A (including taxons 3 and 4) based on ITS sequence data. Bold branches indicate posterior probability values ≥ 0.9. The MP/ML bootstrap support values ≥ 70% are recorded at the nodes. T, ex-type isolates.

Supplementary Figure 5 Phylogram of Lineage A (including taxons 3 and 4) based on Tub2 sequence data. Bold branches indicate posterior probability values ≥ 0.9. The MP/ML bootstrap support values ≥ 70% are recorded at the nodes. T, ex-type isolates.

Supplementary Figure 6 Phylogram of *Ophiostoma ips* complex (including taxon 5) based on combined (ITS + Tub2) sequence data. Bold branches indicate posterior probability values ≥ 0.9. The MP/ML bootstrap support values ≥ 70% are recorded at the nodes. T, ex-type isolates.

Supplementary Figure 7 Phylogram of *Ophiostoma ips* complex (including taxon 5) based on ITS sequence data. Bold branches indicate posterior probability values ≥ 0.9. The MP/ML bootstrap support values ≥ 70% are recorded at the nodes. T, ex-type isolates.

Supplementary Figure 8 Phylogram of *Ophiostoma ips* complex (including taxon 5) based on Tub2 sequence data. Bold branches indicate posterior probability values ≥ 0.9. The MP/ML bootstrap support values ≥ 70% are recorded at the nodes. T, ex-type isolates.

Supplementary Figure 9 Phylogram of *G. cainii* lineage (including taxons 6 and 7) based on Tub2 sequence data. Bold branches indicate posterior probability values ≥ 0.9. The MP/ML bootstrap support values ≥ 70% are recorded at the nodes. T, ex-type isolates.

Supplementary Figure 10 Phylogram of *G. cainii* lineage (including taxons 6 and 7) based on TEF1-α sequence data. Bold branches indicate posterior probability values ≥ 0.9. The MP/ML bootstrap support values ≥ 70% are recorded at the nodes. T, ex-type isolates.

Supplementary Figure 11 Phylogram of *Endoconidiophora* (including taxon 8) based on Tub1 sequence data. Bold branches indicate posterior probability values ≥ 0.9. The MP/ML bootstrap support values ≥ 70% are recorded at the nodes. *D*., *Dendroctonus*; *I*., *Ips*; *L*., *Larix*; *P*., *Picea*.
