# Supplementary figures and images for: Abundance and Diversity of Ophiostomatoid Fungi Associated With the Great Spruce Bark Beetle (Dendroctonus micans) in the Northeastern Qinghai-Tibet Plateau
Source: Front Microbiol. 2021 Oct 18;12:721395. doi: 10.3389/fmicb.2021.721395 (PMC8558629; doi:10.3389/fmicb.2021.721395)

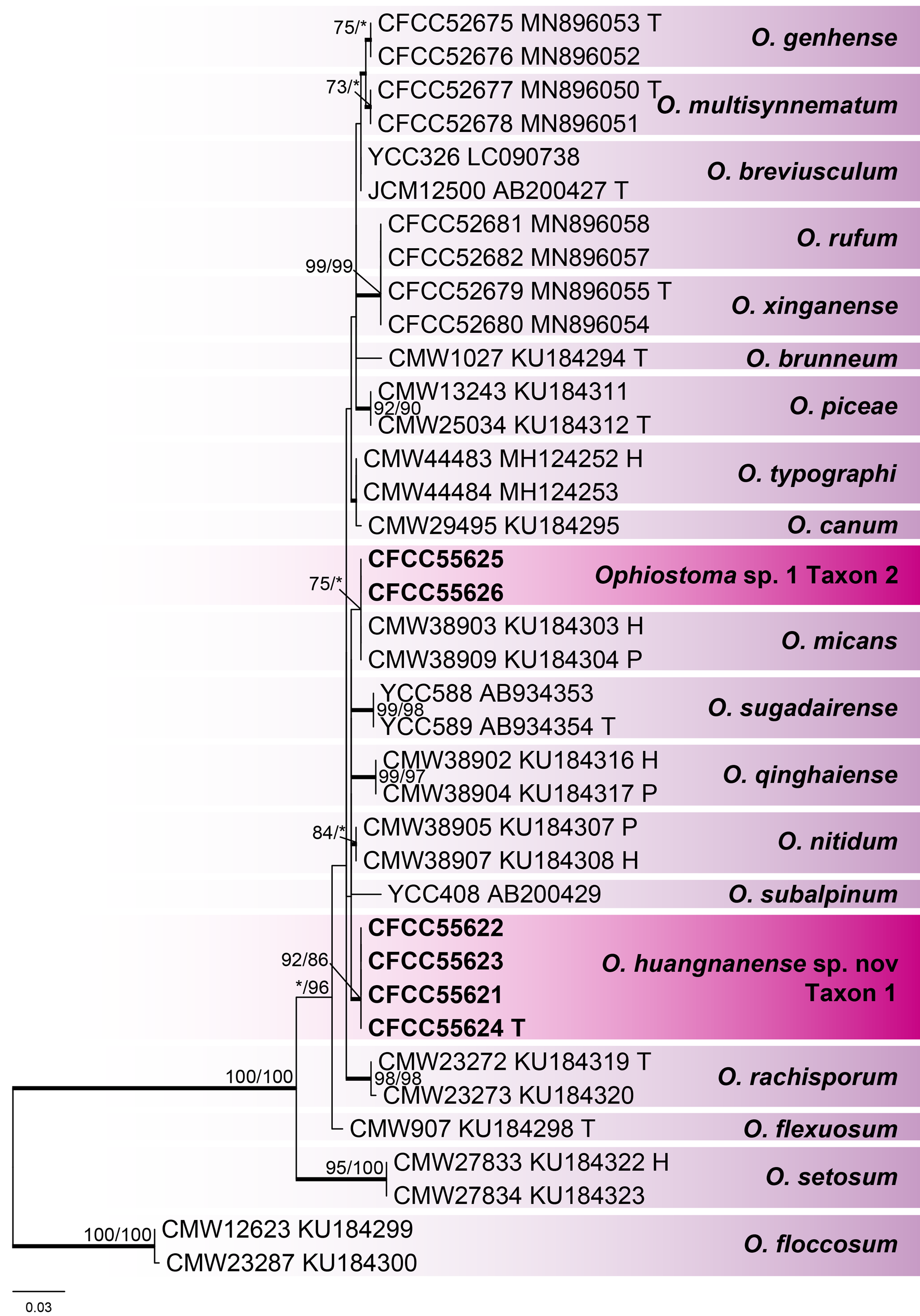

Supplement: Supplementary Figure 1 — Phylogram of Ophiostoma piceae complex (including taxons 1 and 2) based on Tub2 sequence data. Bold branches indicate posterior probability values ≥ 0.9. The MP/ML bootstrap support values ≥ 70% are recorded at the nodes. H, ex-holotype; P, ex-paratype; T, ex-type isolates. [file Image_1.TIF]

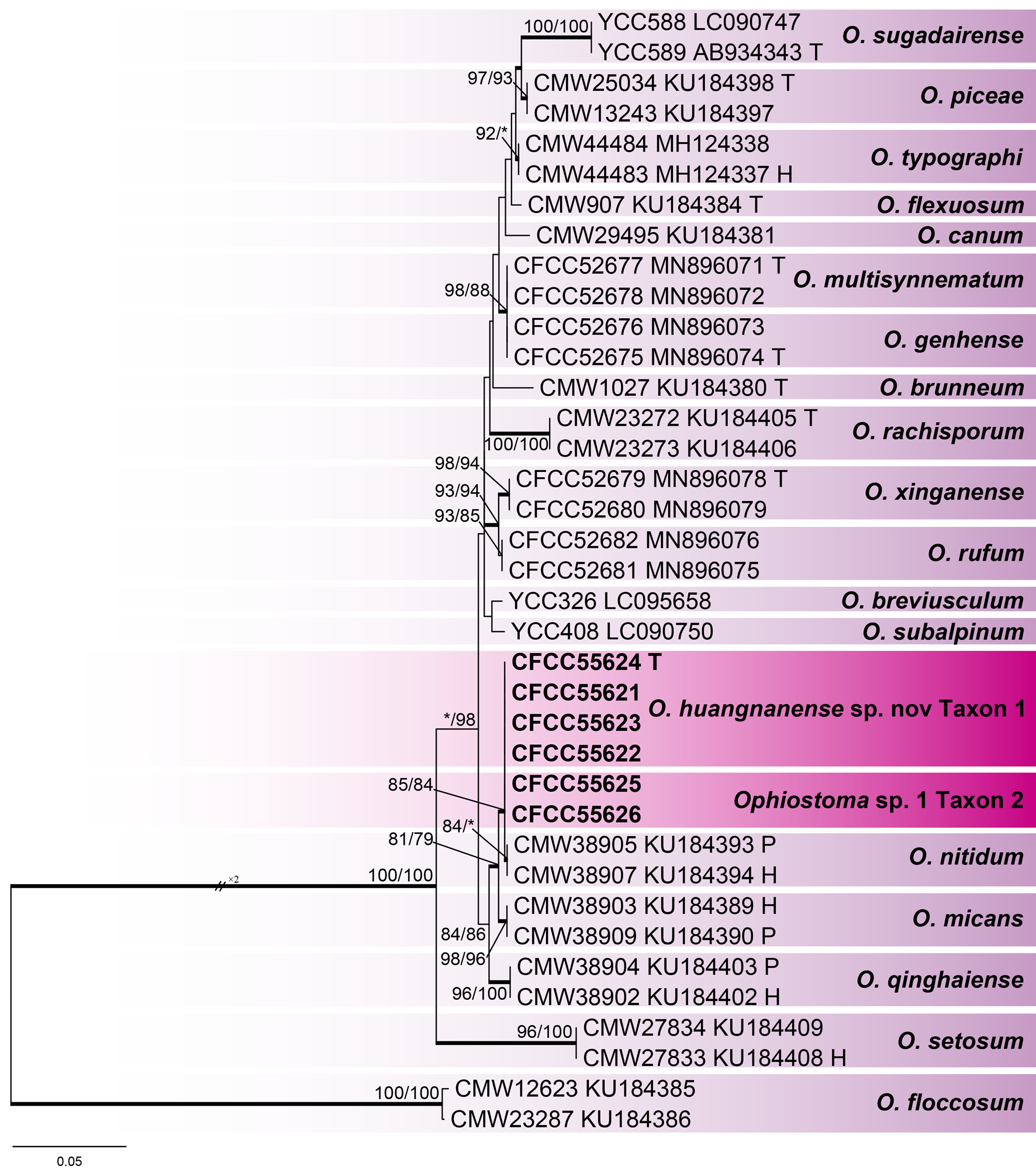

Supplement: Supplementary Figure 2 — Phylogram of Ophiostoma piceae complex (including taxons 1 and 2) based on TEF1-α sequence data. Bold branches indicate posterior probability values ≥ 0.9. The MP/ML bootstrap support values ≥ 70% are recorded at the nodes. H, ex-holotype; P, ex-paratype; T, ex-type isolates. [file Image_2.TIF]

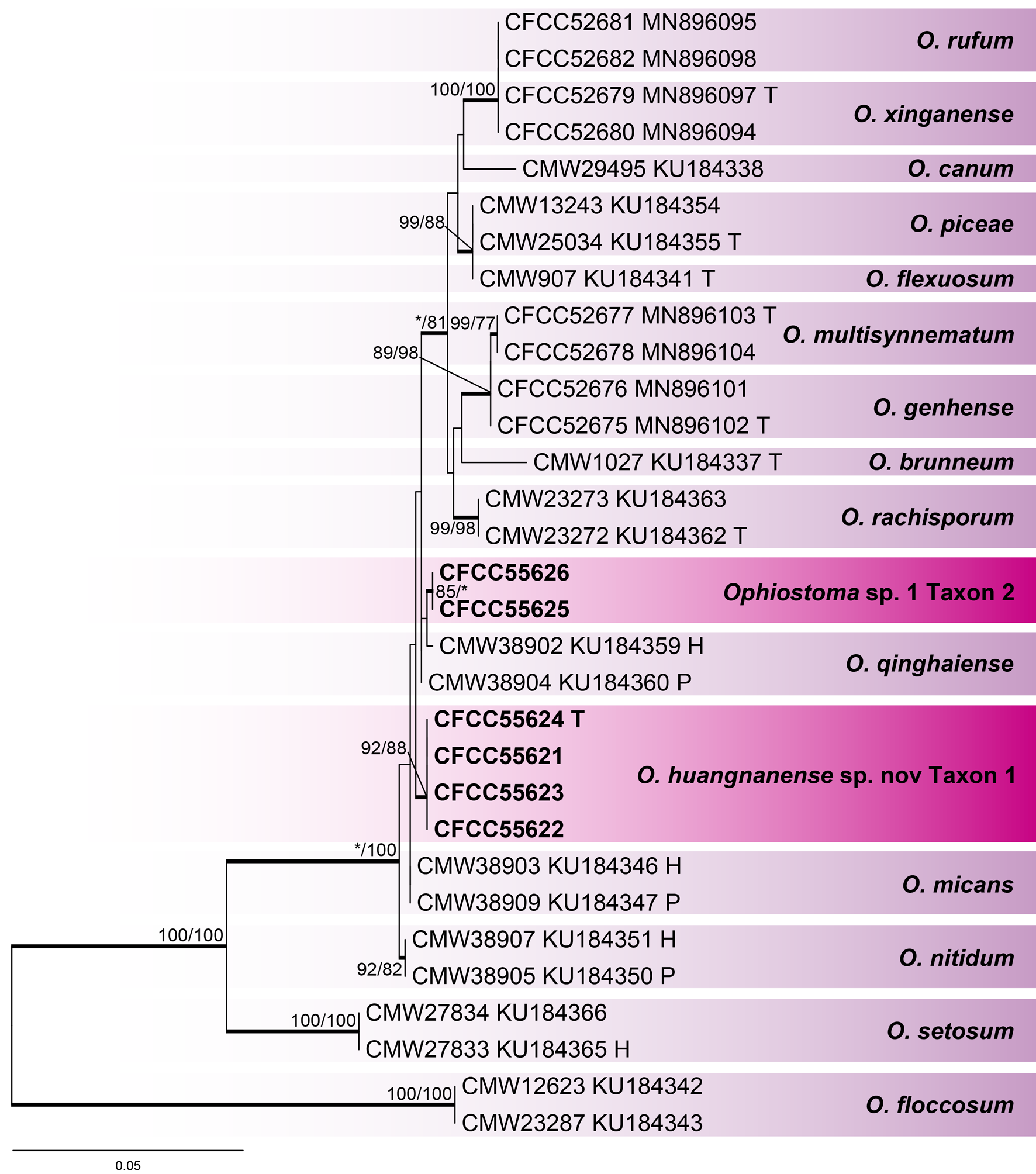

Supplement: Supplementary Figure 3 — Phylogram of Ophiostoma piceae complex (including taxons 1 and 2) based on CAL sequence data. Bold branches indicate posterior probability values ≥ 0.9. The MP/ML bootstrap support values ≥ 70% are recorded at the nodes. H, ex-holotype; P, ex-paratype; T, ex-type isolates. [file Image_3.TIF]

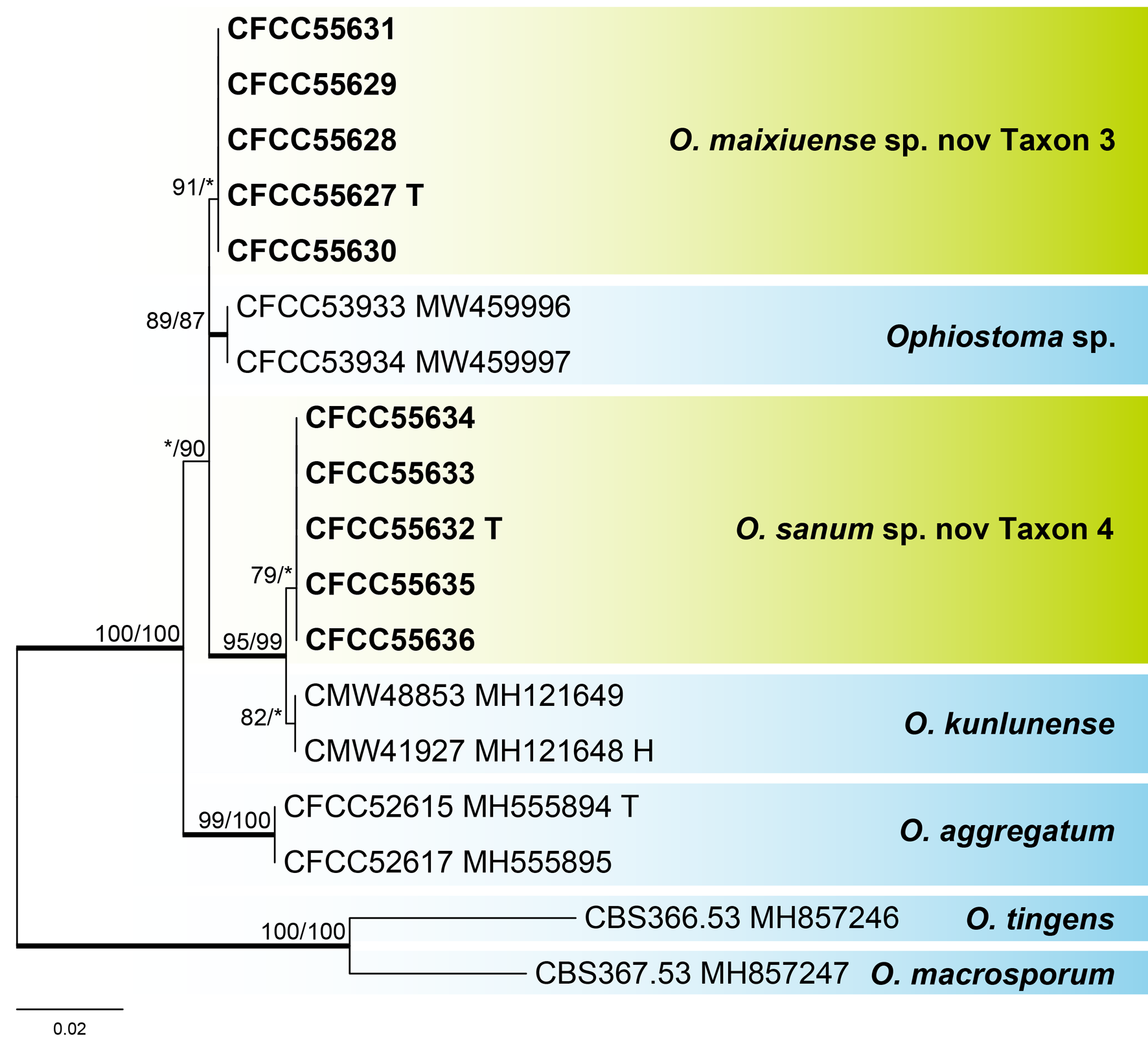

Supplement: Supplementary Figure 4 — Phylogram of Lineage A (including taxons 3 and 4) based on ITS sequence data. Bold branches indicate posterior probability values ≥ 0.9. The MP/ML bootstrap support values ≥ 70% are recorded at the nodes. T, ex-type isolates. [file Image_4.TIF]

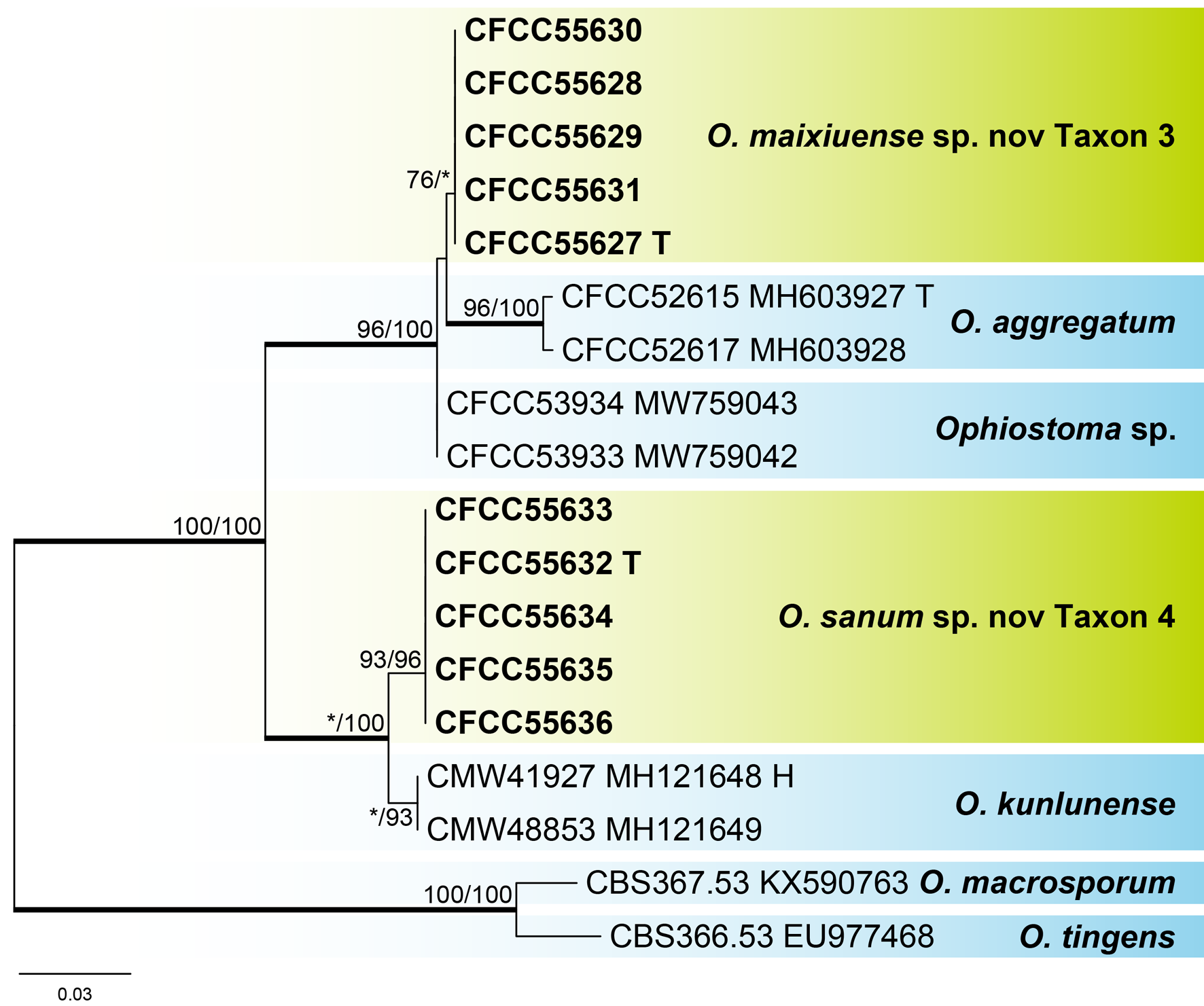

Supplement: Supplementary Figure 5 — Phylogram of Lineage A (including taxons 3 and 4) based on Tub2 sequence data. Bold branches indicate posterior probability values ≥ 0.9. The MP/ML bootstrap support values ≥ 70% are recorded at the nodes. T, ex-type isolates. [file Image_5.TIF]

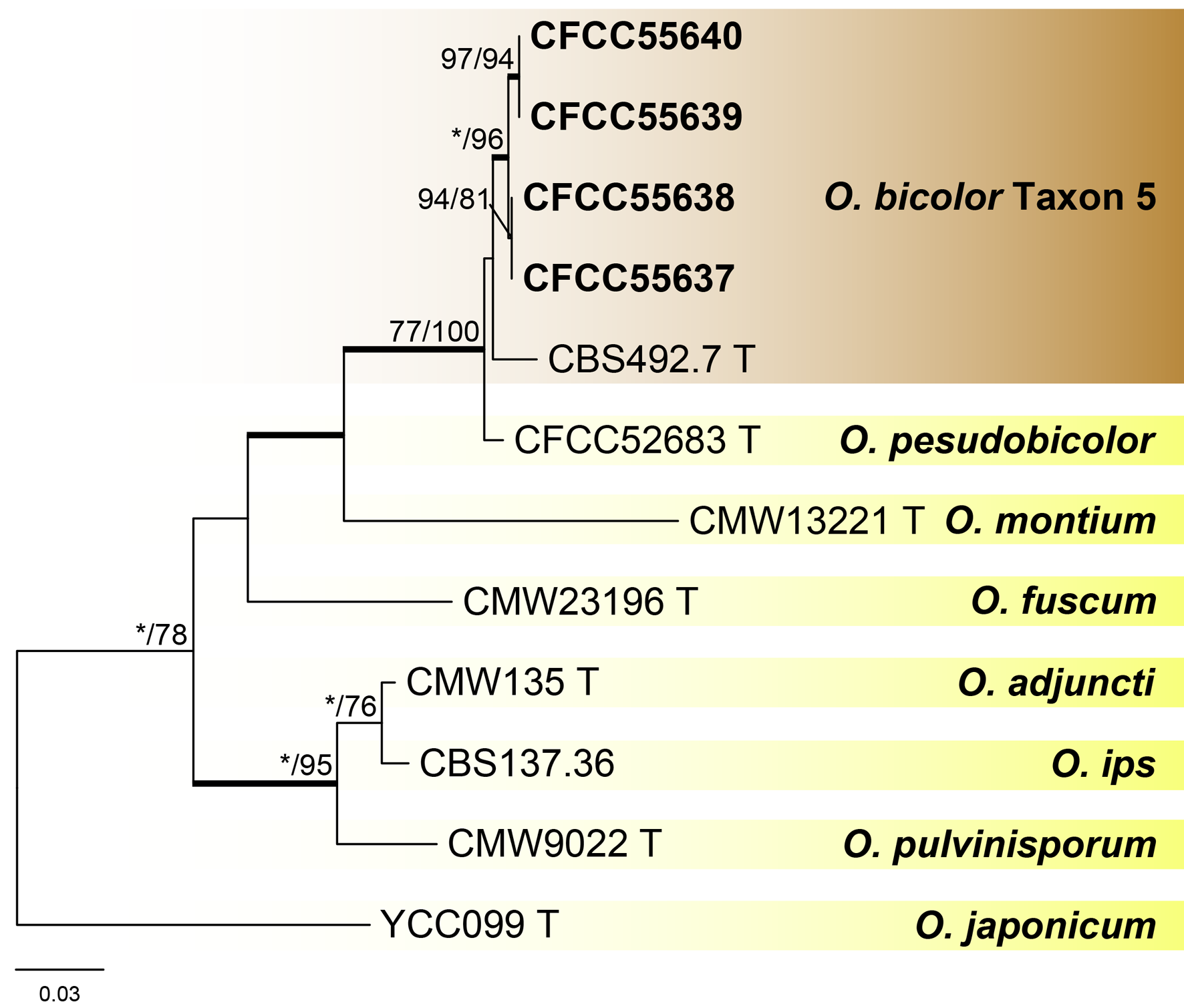

Supplement: Supplementary Figure 6 — Phylogram of Ophiostoma ips complex (including taxon 5) based on combined (ITS + Tub2) sequence data. Bold branches indicate posterior probability values ≥ 0.9. The MP/ML bootstrap support values ≥ 70% are recorded at the nodes. T, ex-type isolates. [file Image_6.TIF]

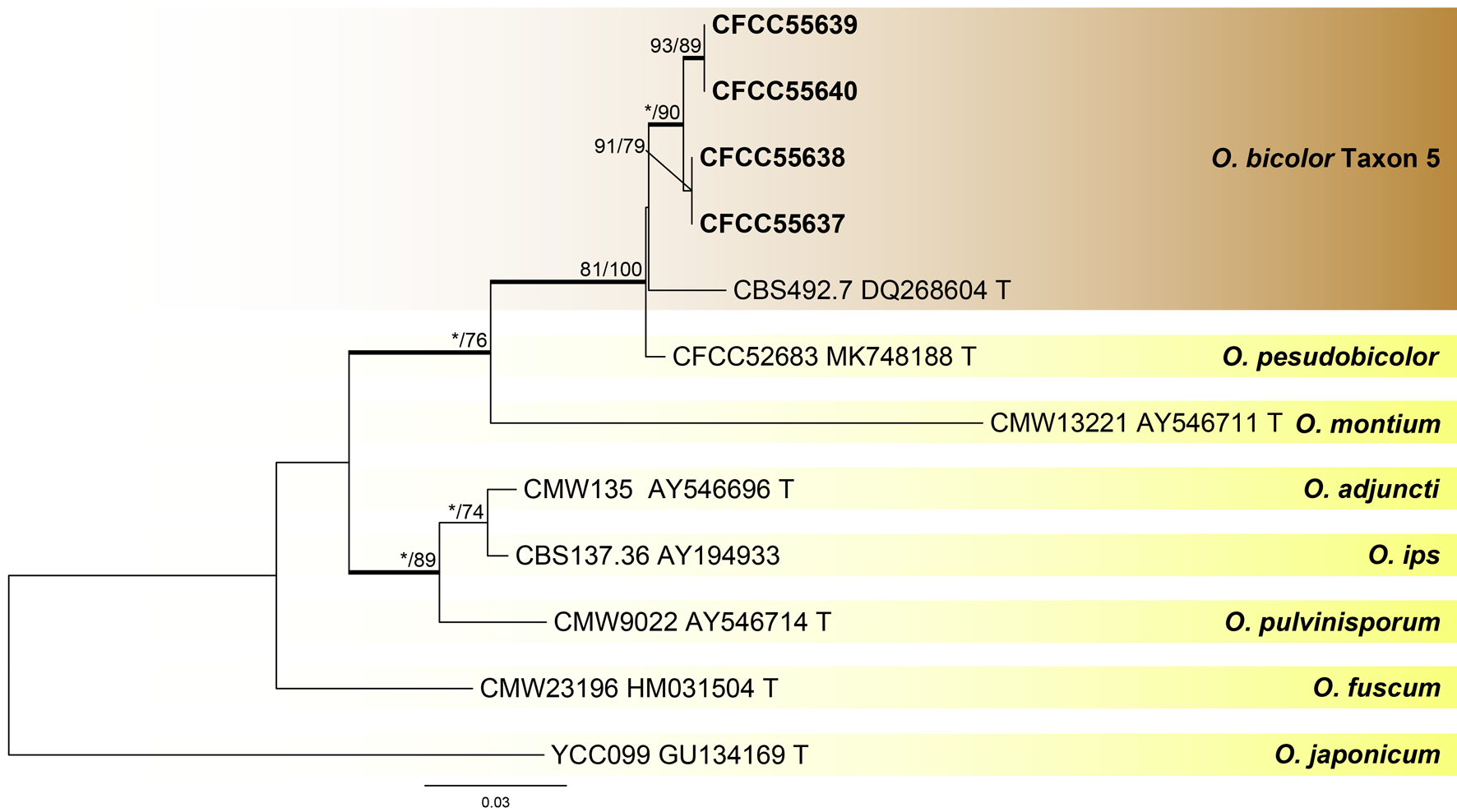

Supplement: Supplementary Figure 7 — Phylogram of Ophiostoma ips complex (including taxon 5) based on ITS sequence data. Bold branches indicate posterior probability values ≥ 0.9. The MP/ML bootstrap support values ≥ 70% are recorded at the nodes. T, ex-type isolates. [file Image_7.TIF]

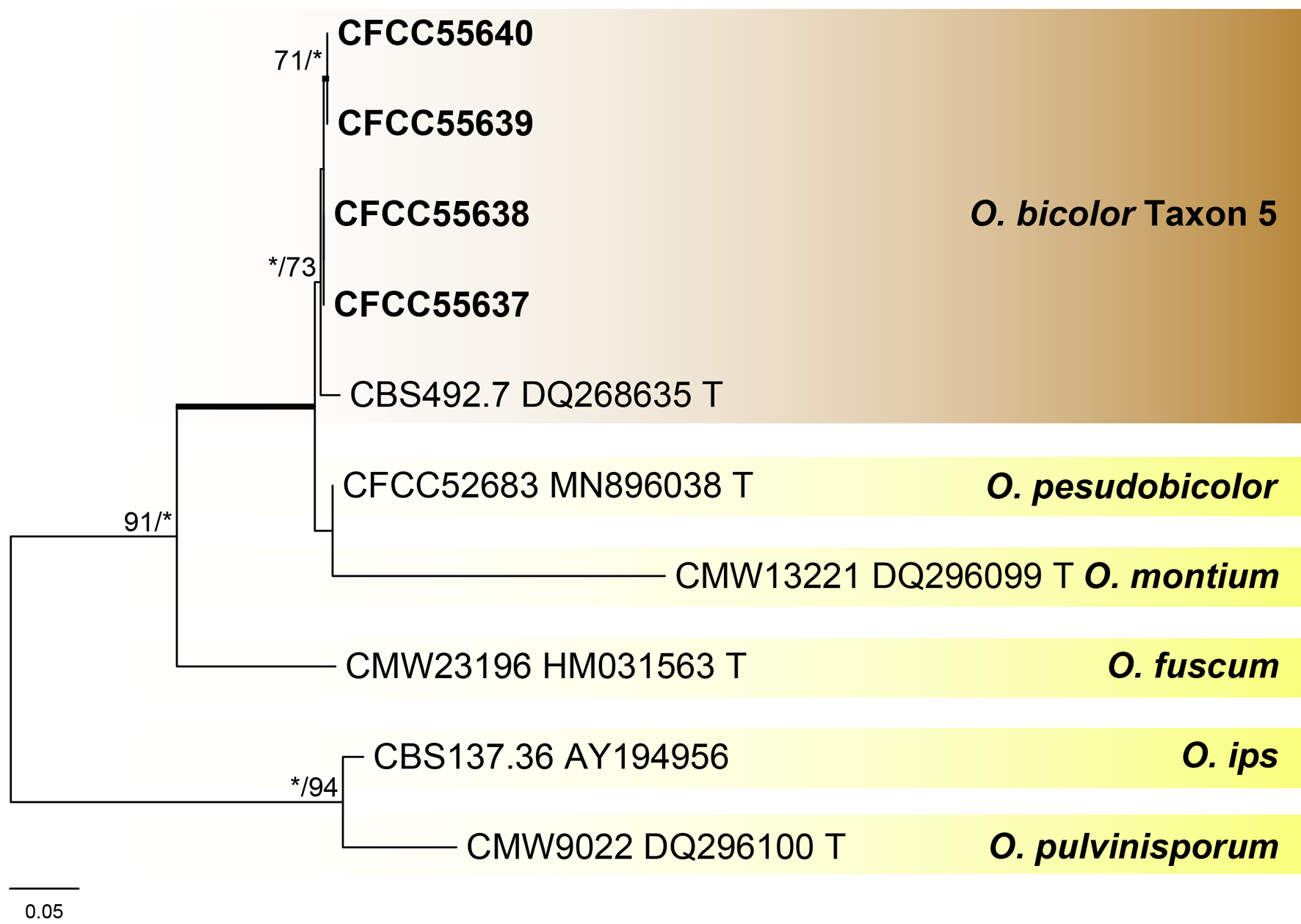

Supplement: Supplementary Figure 8 — Phylogram of Ophiostoma ips complex (including taxon 5) based on Tub2 sequence data. Bold branches indicate posterior probability values ≥ 0.9. The MP/ML bootstrap support values ≥ 70% are recorded at the nodes. T, ex-type isolates. [file Image_8.TIF]

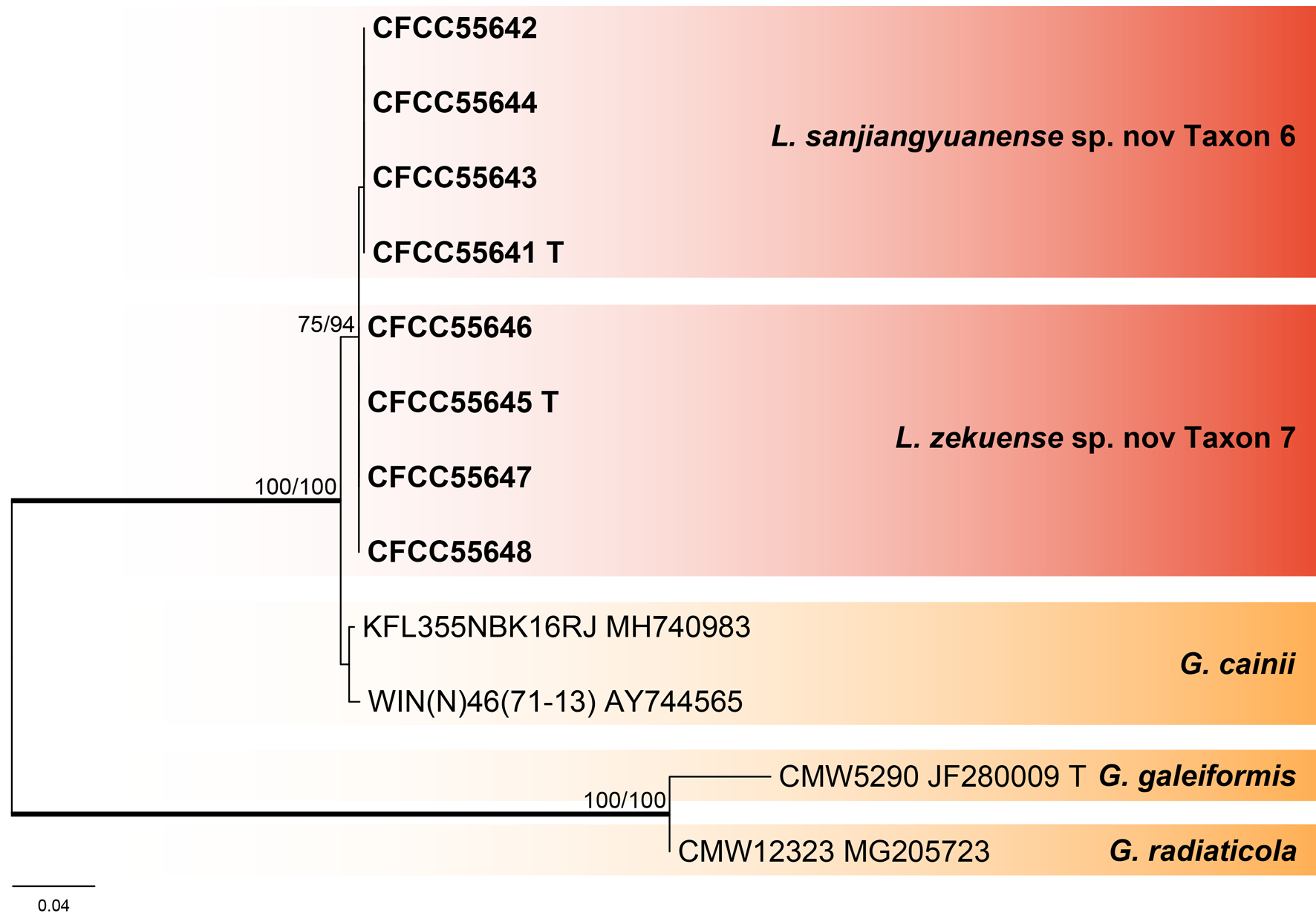

Supplement: Supplementary Figure 9 — Phylogram of G. cainii lineage (including taxons 6 and 7) based on Tub2 sequence data. Bold branches indicate posterior probability values ≥ 0.9. The MP/ML bootstrap support values ≥ 70% are recorded at the nodes. T, ex-type isolates. [file Image_9.TIF]

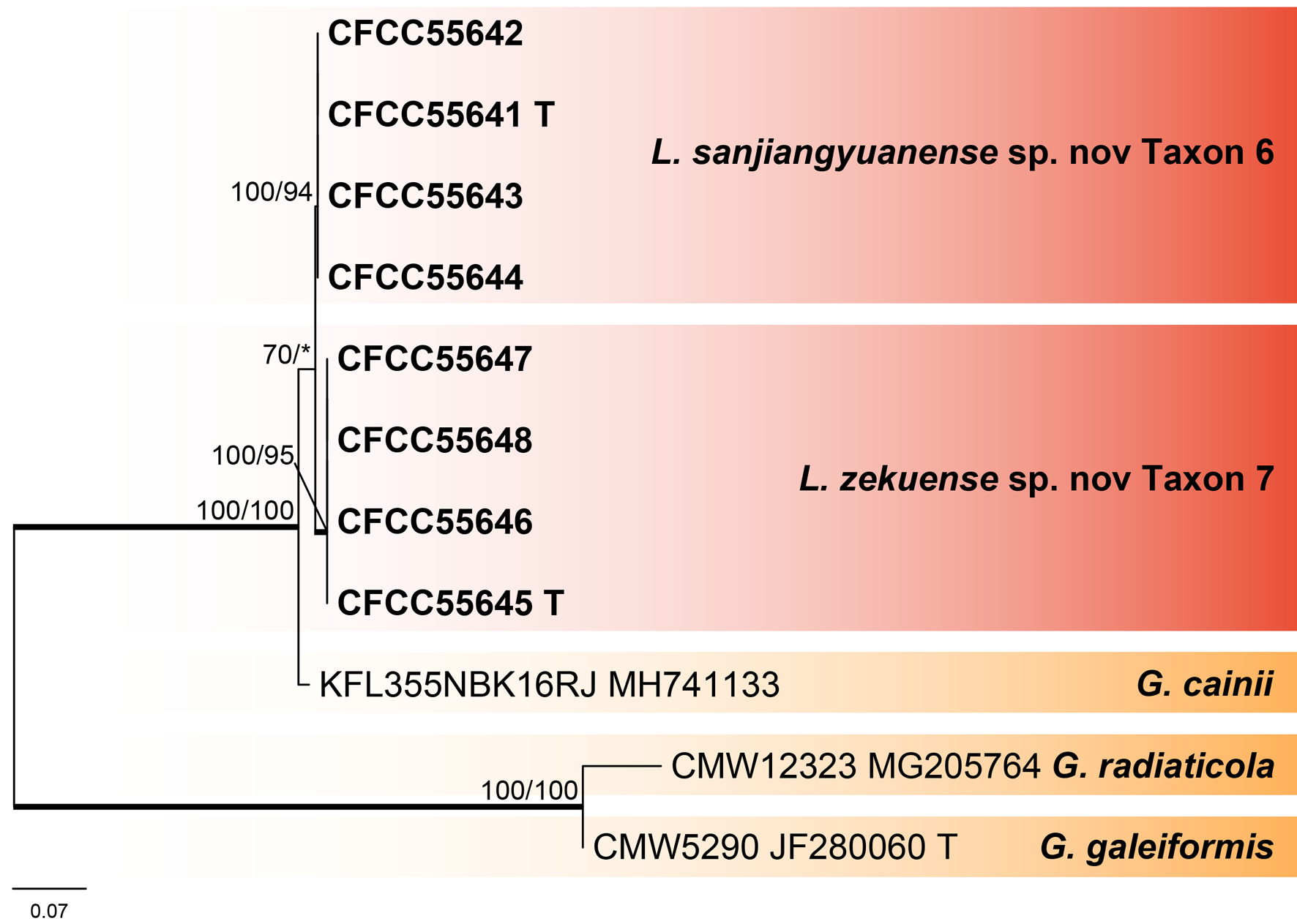

Supplement: Supplementary Figure 10 — Phylogram of G. cainii lineage (including taxons 6 and 7) based on TEF1-α sequence data. Bold branches indicate posterior probability values ≥ 0.9. The MP/ML bootstrap support values ≥ 70% are recorded at the nodes. T, ex-type isolates. [file Image_10.TIF]

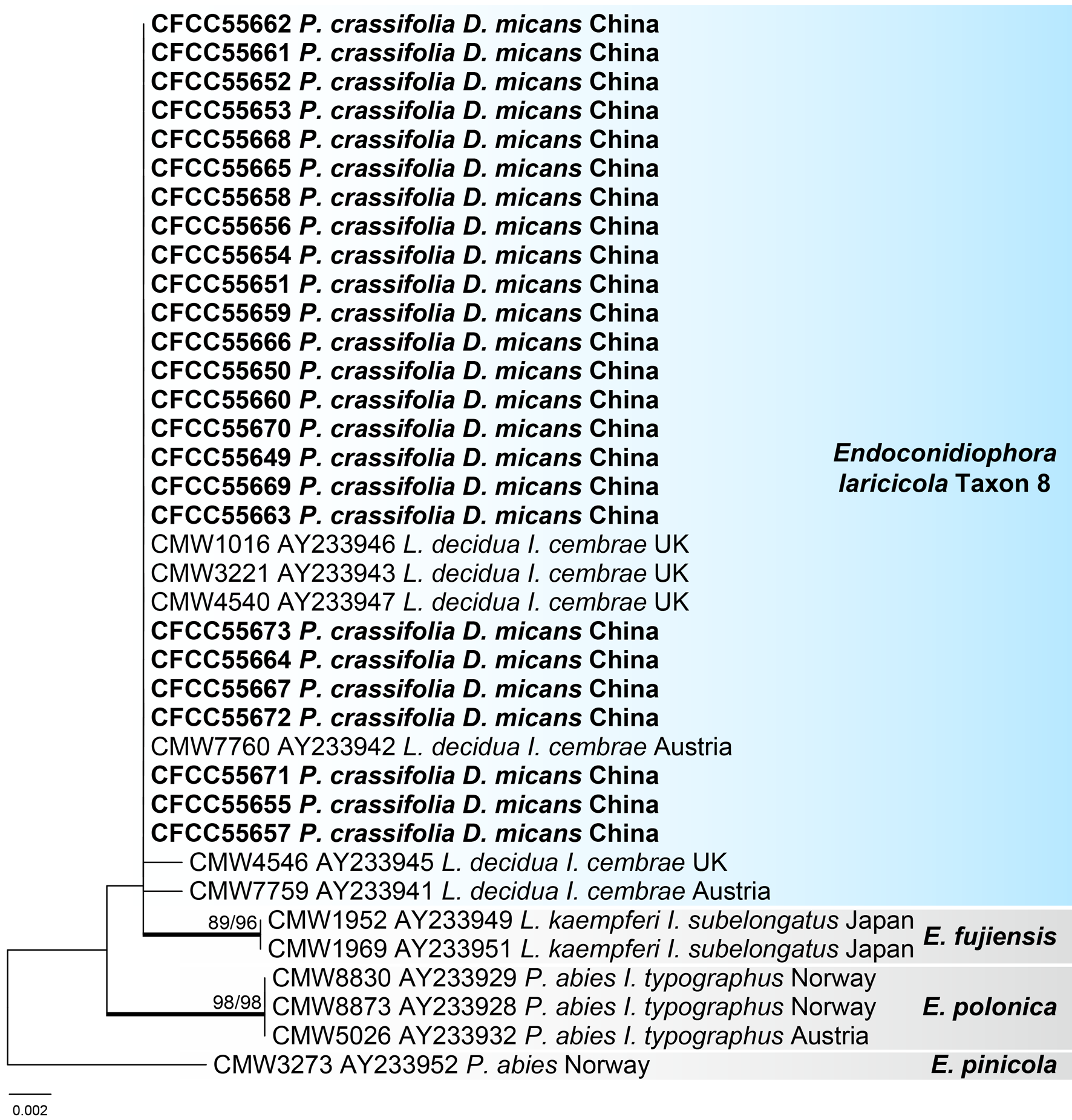

Supplement: Supplementary Figure 11 — Phylogram of Endoconidiophora (including taxon 8) based on Tub1 sequence data. Bold branches indicate posterior probability values ≥ 0.9. The MP/ML bootstrap support values ≥ 70% are recorded at the nodes. D., Dendroctonus; I., Ips; L., Larix; P., Picea. [file Image_11.TIF]
