# Supplementary material for: Abundance and Diversity of Ophiostomatoid Fungi Associated With the Great Spruce Bark Beetle (Dendroctonus micans) in the Northeastern Qinghai-Tibet Plateau
Source: Front Microbiol. 2021 Oct 18;12:721395. doi: 10.3389/fmicb.2021.721395 (PMC8558629; doi:10.3389/fmicb.2021.721395)
Supplement: Supplementary file 12 [file Table_1.DOCX]

**Table S1** The representative strains of ophiostomatoid fungi associated with *Dendroctonus micans* infesting *Picea crassifolia* obtained in Qinghai in this study

| Taxon | Species^1^ | CFCC No.^2^ | CXY No.^3,4^ | Location | GPS information | GenBank number^5^ | | | |
| --- | --- | --- | --- | --- | --- | --- | --- | --- | --- |
|  |  |  |  |  |  | ITS/ITS2-LSU/60S | Tub2/Tub1 | TEF1-α | CAL |
| Ophiostomatales | |  |  |  |  |  |  |  |  |
| *Ophiostoma* | |  |  |  |  |  |  |  |  |
| 1 | ***O. huangnanense*** | 55621 | 3001 | Zeku County, Qinghai, China | N: 35°15'49″ E: 101°53''47″ 3163m | - | MZ327808 | MZ327861 | MZ327802 |
|  |  | 55622 | 3002 | Zeku County, Qinghai, China | N: 35°15'49″ E: 101°53''47″ 3163m | - | MZ327809 | MZ327862 | MZ327803 |
|  |  | 55623 | 3003 | Zeku County, Qinghai, China | N: 35°15'49″ E: 101°53''47″ 3163m | - | MZ327810 | MZ327863 | MZ327804 |
|  |  | 55624 | 3004 T | Zeku County, Qinghai, China | N: 35°15'49″ E: 101°53''47″ 3163m | MZ328850 | MZ327811 | MZ327864 | MZ327805 |
| 2 | Ophiostoma sp. 1 | 55625 | 3005 | Zeku County, Qinghai, China | N: 35°15'49″ E: 101°53''47″ 3163m | MZ328851 | MZ327812 | MZ327865 | MZ327806 |
|  |  | 55626 | 3006 | Zeku County, Qinghai, China | N: 35°15'49″ E: 101°53''47″ 3163m | - | MZ327813 | MZ327866 | MZ327807 |
| 3 | ***O. maixiuense*** | 55627 | 3007 T | Zeku County, Qinghai, China | N: 35°15'49″ E: 101°53''47″ 3163m | MZ328852 | MZ327814 | - | - |
|  |  | 55628 | 3008 | Zeku County, Qinghai, China | N: 35°15'49″ E: 101°53''47″ 3163m | MZ328853 | MZ327815 | - | - |
|  |  | 55629 | 3009 | Zeku County, Qinghai, China | N: 35°15'49″ E: 101°53''47″ 3163m | MZ328854 | MZ327816 | - | - |
|  |  | 55630 | 3010 | Zeku County, Qinghai, China | N: 35°15'49″ E: 101°53''47″ 3163m | MZ328855 | MZ327817 | - | - |
|  |  | 55631 | 3011 | Zeku County, Qinghai, China | N: 35°15'49″ E: 101°53''47″ 3163m | MZ328856 | MZ327818 | - | - |
| 4 | ***O. sanum*** | 55632 | 3012 T | Zeku County, Qinghai, China | N: 35°15'49″ E: 101°53''47″ 3163m | MZ328857 | MZ327819 | - | - |
|  |  | 55633 | 3013 | Zeku County, Qinghai, China | N: 35°15'49″ E: 101°53''47″ 3163m | MZ328858 | MZ327820 | - | - |
|  |  | 55634 | 3014 | Zeku County, Qinghai, China | N: 35°15'49″ E: 101°53''47″ 3163m | MZ328859 | MZ327821 | - | - |
|  |  | 55635 | 3015 | Zeku County, Qinghai, China | N: 35°15'49″ E: 101°53''47″ 3163m | MZ328860 | MZ327822 | - | - |
|  |  | 55636 | 3016 | Zeku County, Qinghai, China | N: 35°15'49″ E: 101°53''47″ 3163m | MZ328861 | MZ327823 | - | - |
| 5 | *O. bicolor* | 55637 | 3017 | Menyuan County, Qinghai, China | N: 38°14'39″ E: 100°14''45″ 2942m | MZ328862 | MZ327824 | - | - |
|  |  | 55638 | 3018 | Menyuan County, Qinghai, China | N: 38°14'39″ E: 100°14''45″ 2942m | MZ328863 | MZ327825 | - | - |
|  |  | 55639 | 3019 | Qilian County, Qinghai, China | N: 37°6'45″ E: 102°29''56″ 2964m | MZ328864 | MZ327826 | - | - |
|  |  | 55640 | 3020 | Qilian County, Qinghai, China | N: 37°6'45″ E: 102°29''56″ 2964m | MZ328865 | MZ327827 | - | - |
| *Leptographium* | |  |  |  |  |  |  |  |  |
| 6 | ***L. sanjiangyuanense*** | 55641 | 3021 T | Zeku County, Qinghai, China | N: 35°15'49″ E: 101°53''47″ 3163m | MZ327900 | MZ327828 | MZ327867 | - |
|  |  | 55642 | 3022 | Zeku County, Qinghai, China | N: 35°15'49″ E: 101°53''47″ 3163m | - | MZ327829 | MZ327868 | - |
|  |  | 55643 | 3023 | Zeku County, Qinghai, China | N: 35°15'49″ E: 101°53''47″ 3163m | - | MZ327830 | MZ327869 | - |
|  |  | 55644 | 3024 | Zeku County, Qinghai, China | N: 35°15'49″ E: 101°53''47″ 3163m | - | MZ327831 | MZ327870 | - |
| 7 | ***L. zekuense*** | 55645 | 3025 T | Zeku County, Qinghai, China | N: 35°15'49″ E: 101°53''47″ 3163m | MZ327901 | MZ327832 | MZ327871 | - |
|  |  | 55646 | 3026 | Zeku County, Qinghai, China | N: 35°15'49″ E: 101°53''47″ 3163m | - | MZ327833 | MZ327872 | - |
|  |  | 55647 | 3027 | Zeku County, Qinghai, China | N: 35°15'49″ E: 101°53''47″ 3163m | - | MZ327834 | MZ327873 | - |
|  |  | 55648 | 3028 | Zeku County, Qinghai, China | N: 35°15'49″ E: 101°53''47″ 3163m | - | MZ327835 | MZ327874 | - |
| Microascales | |  |  |  |  |  |  |  |  |
| *Endoconidiophora* | |  |  |  |  |  |  |  |  |
| 8 | *E. laricicola* | 55649 | 3029 | Zeku County, Qinghai, China | N: 35°15'49″ E: 101°53''47″ 3163m | MZ327875 | MZ327836 | - | - |
|  |  | 55650 | 3030 | Zeku County, Qinghai, China | N: 35°15'49″ E: 101°53''47″ 3163m | MZ327876 | MZ327837 | - | - |
|  |  | 55651 | 3031 | Zeku County, Qinghai, China | N: 35°15'49″ E: 101°53''47″ 3163m | MZ327877 | MZ327838 | - | - |
|  |  | 55652 | 3032 | Zeku County, Qinghai, China | N: 35°15'49″ E: 101°53''47″ 3163m | MZ327878 | MZ327839 | - | - |
|  |  | 55653 | 3033 | Zeku County, Qinghai, China | N: 35°15'49″ E: 101°53''47″ 3163m | MZ327879 | MZ327840 | - | - |
|  |  | 55654 | 3034 | Zeku County, Qinghai, China | N: 35°15'49″ E: 101°53''47″ 3163m | MZ327880 | MZ327841 | - | - |
|  |  | 55655 | 3035 | Zeku County, Qinghai, China | N: 35°15'49″ E: 101°53''47″ 3163m | MZ327881 | MZ327842 | - | - |
|  |  | 55656 | 3036 | Zeku County, Qinghai, China | N: 35°15'49″ E: 101°53''47″ 3163m | MZ327882 | MZ327843 | - | - |
|  |  | 55657 | 3037 | Zeku County, Qinghai, China | N: 35°15'49″ E: 101°53''47″ 3163m | MZ327883 | MZ327844 | - | - |
|  |  | 55658 | 3038 | Zeku County, Qinghai, China | N: 35°15'49″ E: 101°53''47″ 3163m | MZ327884 | MZ327845 | - | - |
|  |  | 55659 | 3039 | Zeku County, Qinghai, China | N: 35°15'49″ E: 101°53''47″ 3163m | MZ327885 | MZ327846 | - | - |
|  |  | 55660 | 3040 | Zeku County, Qinghai, China | N: 35°15'49″ E: 101°53''47″ 3163m | MZ327886 | MZ327847 | - | - |
|  |  | 55661 | 3041 | Zeku County, Qinghai, China | N: 35°15'49″ E: 101°53''47″ 3163m | MZ327887 | MZ327848 | - | - |
|  |  | 55662 | 3042 | Zeku County, Qinghai, China | N: 35°15'49″ E: 101°53''47″ 3163m | MZ327888 | MZ327849 | - | - |
|  |  | 55663 | 3043 | Zeku County, Qinghai, China | N: 35°15'49″ E: 101°53''47″ 3163m | MZ327889 | MZ327850 | - | - |
|  |  | 55664 | 3044 | Zeku County, Qinghai, China | N: 35°15'49″ E: 101°53''47″ 3163m | MZ327890 | MZ327851 | - | - |
|  |  | 55665 | 3045 | Zeku County, Qinghai, China | N: 35°15'49″ E: 101°53''47″ 3163m | MZ327891 | MZ327852 | - | - |
|  |  | 55666 | 3046 | Zeku County, Qinghai, China | N: 35°15'49″ E: 101°53''47″ 3163m | MZ327892 | MZ327853 | - | - |
|  |  | 55667 | 3047 | Zeku County, Qinghai, China | N: 35°15'49″ E: 101°53''47″ 3163m | MZ327893 | MZ327854 | - | - |
|  |  | 55668 | 3048 | Zeku County, Qinghai, China | N: 35°15'49″ E: 101°53''47″ 3163m | MZ327894 | MZ327855 | - | - |
|  |  | 55669 | 3049 | Zeku County, Qinghai, China | N: 35°15'49″ E: 101°53''47″ 3163m | MZ327895 | MZ327856 | - | - |
|  |  | 55670 | 3050 | Zeku County, Qinghai, China | N: 35°15'49″ E: 101°53''47″ 3163m | MZ327896 | MZ327857 | - | - |
|  |  | 55671 | 3051 | Zeku County, Qinghai, China | N: 35°15'49″ E: 101°53''47″ 3163m | MZ327897 | MZ327858 | - | - |
|  |  | 55672 | 3052 | Zeku County, Qinghai, China | N: 35°15'49″ E: 101°53''47″ 3163m | MZ327898 | MZ327859 | - | - |
|  |  | 55673 | 3053 | Zeku County, Qinghai, China | N: 35°15'49″ E: 101°53''47″ 3163m | MZ327899 | MZ327860 | - | - |

^1^Species names in bold are novel species described in this study.

^2^CFCC: China Forestry Culture Collection Center, Beijing, China.

^3^CXY (Culture Xingyao): Culture collection of the Research Institute of Forest Ecology, Environment and Protection, Chinese Academy of Forestry.

^4^T = ex-holotype isolate.

^5^ITS. the internal transcribed spacer regions 1 and 2 of the nuclear ribosomal DNA operon, including the 5.8S region; ITS2-LSU. the internal transcribed spacer 2 and part of the 28S of the rDNA operon; Tub1. the β-tubulin gene region (Tub1); Tub2: the β-tubulin gene region (Tub2); TEF1-α. the transcription elongation factor 1-α gene region; CAL. the calmodulin gene region; 60S. the partial 60S ribosomal protein RPL10 gene.
