# Supplementary material for: Abundance and Diversity of Ophiostomatoid Fungi Associated With the Great Spruce Bark Beetle (Dendroctonus micans) in the Northeastern Qinghai-Tibet Plateau
Source: Front Microbiol. 2021 Oct 18;12:721395. doi: 10.3389/fmicb.2021.721395 (PMC8558629; doi:10.3389/fmicb.2021.721395)
Supplement: Supplementary file 13 [file Table_2.DOCX]

**Table S2** Diversity and abundance of ophiostomatoid strains collected during the invesitigation of this study

| Genus | Taxon | Species | Numbers of isolates | | Total | Total percentage |
| --- | --- | --- | --- | --- | --- | --- |
|  |  |  | Beetles | Galleries |  |  |
| Ophiostomatales |  |  |  |  |  |  |
| *Ophiostoma* | 1 | *O. huangnanense* | 24 | 0 | 24 | 10.91% |
|  | 2 | *Ophiostoma* sp. 1 | 0 | 3 | 3 | 1.36% |
|  | 3 | *O. maixiuense* | 0 | 15 | 15 | 6.82% |
|  | 4 | *O. sanum* | 15 | 9 | 24 | 10.91% |
|  | 5 | *O. bicolor* | 0 | 18 | 18 | 8.18% |
| *Leptographium* | 6 | *L. sanjiangyuanense* | 0 | 36 | 36 | 16.36% |
|  | 7 | *L. zekuense* | 10 | 0 | 10 | 4.55% |
| Microascales |  |  |  |  |  |  |
| *Endoconidiophora* | 8 | *E. laricicola* | 90 | 0 | 90 | 40.91% |
| Total |  |  | 147 | 73 | 220 | 100.00% |
