# Supplementary material for: Abundance and Diversity of Ophiostomatoid Fungi Associated With the Great Spruce Bark Beetle (Dendroctonus micans) in the Northeastern Qinghai-Tibet Plateau
Source: Front Microbiol. 2021 Oct 18;12:721395. doi: 10.3389/fmicb.2021.721395 (PMC8558629; doi:10.3389/fmicb.2021.721395)
Supplement: Supplementary file 14 [file Table_3.DOCX]

**Table S3** Nucleotide substitution models obtained from jModelTest v. 2.1.7

| Genus | Group | dataset | Nucleotide substitution models |
| --- | --- | --- | --- |
| *Ophiostoma* |  | ITS | GTR+I+G |
|  | *O. piceae* complex | Tub2 | GTR+G |
|  |  | TEF1-α | GTR+G |
|  |  | CAL | GTR+G |
|  |  | combined | GTR+I+G |
|  | Lineage A | ITS | HKY+I |
|  |  | Tub2 | GTR+G |
|  |  | combined | GTR+I |
|  | *O. ips* comolex | ITS | GTR+G |
|  |  | Tub2 | HKY+I |
|  |  | combined | GTR+G |
| *Leptographium* |  | ITS2-LSU | GTR+I+G |
|  | *G. cainii* lineage | Tub2 | K80+G |
|  |  | TEF1-α | HKY+G |
|  |  | combined | GTR+G |
| *Endoconidiophora* |  | 60S | K80+I |
|  |  | Tub1 | HKY |
